# Functional morphology of parasitic isopods: understanding morphological adaptations of attachment and feeding structures in *Nerocila* as a pre-requisite for reconstructing the evolution of Cymothoidae

**DOI:** 10.7717/peerj.2188

**Published:** 2016-07-05

**Authors:** Christina Nagler, Joachim T. Haug

**Affiliations:** 1 Department of Biology, Ludwig-Maximilians-Universität München, Planegg-Martinsried, Germany; 2 GeoBio-Center, Ludwig-Maximilians-Universität München, Germany

**Keywords:** Cymothoidae, Parasites, Modifications, Adaptations, Mouth cone, Attachment

## Abstract

Parasites significantly influence food webs and ecosystems and occur all over the world in almost every animal group. Within crustaceans there are numerous examples of ectoparasites; for example, representatives of the isopod group Cymothoidae. These obligatory parasitic isopods are relatively poorly studied regarding their functional morphology. Here we present new details of the morphological adaptations to parasitism of the cymothoiid ingroup *Nerocila* with up-to-date imaging methods (macro photography, stereo imaging, fluorescence photography, micro CT, and histology). Central aspects of the study were (1) the morphology of the mouthparts and (2) the attachment on the host, hence the morphology of the thoracopods. The mouthparts (labrum, mandibles, paragnaths, maxillulae, maxillae, maxillipeds) form a distinct mouth cone and are most likely used for true sucking. The mouthparts are tightly “folded” around each other and provide functional rails for the only two moving mouthparts, mandible and maxillula. Both are not moving in an ancestral-type median-lateral movement, but are strongly tilted to move more in a proximal-distal axis. New details concerning the attachment demonstrate that the angular arrangement of the thoracopods is differentiated to impede removal by the host. The increased understanding of morphological adaptation to parasitism of modern forms will be useful in identifying disarticulated (not attached to the host) fossil parasites.

## Introduction

### Parasitic arthropods

Parasites are found in all habitats and most major groups of metazoans (Poulin & Morand, 2000; Zrzavý, 2001). Parasitism strongly influences food webs and represents an important force of evolution due to co-evolutionary aspects of host-parasite relationships (Boucot & Poinar, 2010; Boyko et al., 2013; Dunne et al., 2013; Al-Zubaidy & Mhaisen, 2013; De Baets & Littlewood, 2015; Jephcott et al., 2016). Thus, parasites are both ecologically and evolutionary significant.

Arthropods are often considered to be the most successful groups of organisms in evolutionary terms. Despite the fact that this statement is logically incorrect (see e.g., discussion in Haug, Haug & Garwood, 2016), arthropods indeed are impressive concerning species richness, biomass, individual richness and morphological and ecological diversity. Hence, it is not surprising that also among arthropods numerous groups have evolved parasitic life habits. While indeed most people might think of insects in this respect other, non-insect crustaceans (as insects are derived ingroup crustaceans) are of central interest in this respect.

## Parasitic isopods

Among the numerous non-insect crustacean lineages that have evolved a parasitic lifestyle, different ingroups of Isopoda may be considered especially interesting due to a number of partly interconnected factors: (1) diverse morphology, (2) size and (3) economical impact (e.g., Rohde, 2005; Klompmaker et al., 2014; Klompmaker & Boxshall, 2015).

Concerning (1) numerous lineages of Isopoda have independently evolved different styles of parasitism and hence provide “experimental set-ups” that can be studied for understanding the evolution of parasitism.

With this, it benefits from (2), which means that the to be studied specimens are of comparably large size which makes studies of adaptations to parasitism often easier compared to microscopic organisms. It also means that chances are higher that fossil forms either of the parasite itself or of its traces may be preserved and can be successfully incorporated into an evolutionary reconstruction.

Size also influences (3). Due to the comparably large size, parasitic isopods cause significant economic losses in fisheries and aquaculture, because they reduce their hosts’ fitness by influencing physiology, behavior, morphology and anatomy (Östlund-Nilsson et al., 2005; Roche, Strong & Binning, 2013). Also, the absence of parasitic isopods has been supposed to be usable as a biological indicator for ciguatera (fish poisoning) toxin (Bunkley-Williams, Williams Jr & Bashirullah, 2006).

## The group of interest: Cymothoidae

As already stated, there are numerous lineages within Isopoda that evolved a parasitic lifestyle. Within the species rich group Cymothoida (sensu Brandt & Poore, 2003) there are different groups which exhibiting different types of parasitism among other lifestyles:

– Representatives of Aegidae attach to a host (fish) only for a short time, as long as they consume from it, just to leave the host afterwards (Lester, 2005).
– Representatives of Cymothoidae act relatively similar to aegiids when juveniles (Fogelman & Grutter, 2008) but attach to their host (also a fish) permanently when being adult (e.g., Brusca & Iverson, 1985; Bunkley-Williams& Williams Jr, 1998; Smit, Bruce & Hadfield, 2014; Jones et al., 2008).
– Representatives of Gnathiidae also act in a comparable way to aegiids but only during a specific larval phase (Hispano, Bulto & Blanch, 2014).
– Representatives of Epicaridea (Bopyroidea + Cryptoniscoidea) parasitize on small crustaceans (mostly copepods) during their larval phase and mainly on large crustaceans (decapods) when being adult (Williams & Boyko, 2012).

With this, Cymothoida appears to be an interesting group for studying the evolution of parasitism, including deep time aspects based on fossils. There are different ways for how a parasitic lifestyle can be inferred based on fossils (see Nagler et al., 2016 for discussion). For Cymothoida, parasitic representatives have been identified based on:

– malformations on the host (e.g., by Bopyridae; Klompmaker et al., 2014)
– isolated specimens attributed to a specific group and in comparison to the life-habits of this modern group (Cryptoniscoidea; Serrano-Sánchez et al., 2016).
– direct co-occurrence of parasite on host (Nagler et al., 2016).

Unfortunately, one source for indication of possible parasitic life habits has not been successfully exploited for fossil cymothoidans: the functional morphology. This might be more important for representatives of Cymothoidae than for Bopyridae as they are more likely to be found isolated. On the other hand cymothoiids, as permanently attached forms, should show more pronounced adaptation than the only temporarily attached representatives of Aegidae and Gnathiidae. There are at least two aspects of the functional morphology that are important in this aspect. One is the attachment to the host. In extant forms a forceful removal of a cymothoiid leaves pronounced scars in the skin and tissue of the host (Bunkley-Williams & Williams Jr, 1998). The other should concern the mouthparts. Although it has been suggested that especially insects represent extreme cases of evolutionary derived mouthparts (Blanke et al., 2015), we should also expect strong modification representing true adaptations in cymothoiids. In an evolutionary framework those representatives of Cymothoidae should be most interesting as they are considered to represent less strongly derived forms.

The biology of parasitic isopods is relatively poorly studied despite their remarkable size and their ecological and economical importance in contrast to other crustacean parasites (Bunkley-Williams, Williams Jr & Bashirullah, 2006; Bariche & Trilles, 2006; El-Shahawy & Desouky, 2010; Roche, Strong & Binning, 2013; Smit, Bruce & Hadfield, 2014). While it should be clear that the morphology of cymothoiids is adapted to parasitism (Brandt & Poore, 2003; Wilson, 2009; Smit, Bruce & Hadfield, 2014), details of their specialized functional morphology seem largely unclear. Several authors (e.g., Bunkley-Williams & Williams Jr, 1998; Brandt & Poore, 2003) suggested that a reappraisal of the morphology and biology of cymothoiids is needed.

So far there have been few studies related to the feeding biology of cymothoiid isopods. The most comprehensive work is that of Günther (1931) describing the functional morphology of parasitic cymothoidan isopods and their relatives. He highlighted the differences between non-parasitic forms (Cirolanidae), shortly attached forms (Aegidae) and permanently attached forms of Cymothoidae and described their mouthparts based on microscopic observations.

Unfortunately this study has been largely unnoticed, probably due to the fact that it was published in German. This seems to be the only study so far, and hence our only database for understanding the functional morphology of these parasites. In comparison to fossil material this study is also difficult, because the representation is restricted to line drawings of isolated structures (e.g., Günther, 1931; Thatcher, 1997).

Here we aim at amending our still limited knowledge of the functional morphology of the mouthparts and thoracopods of cymothoiids especially as a basis for a future comparison to fossil material. We studied specimens of the cymothoiid ingroup *Nerocila* with up-to-date imaging techniques. The different techniques used (fluorescence microscopy, microCT, histology) provide a novel perspective and innovative combination of these, particularly in the relationship of the mouthparts to each other. We provide with this study a base for understanding the functional morphology of cymothoiids and their adaptations to parasitism. Furthermore, this study will help to separate between scavenging, predatory or parasitic cymothoidans based on morphological characters alone, especially in fossil, disarticulated cymothoidans.

## Material and Methods

### Material

**Figure 1:**
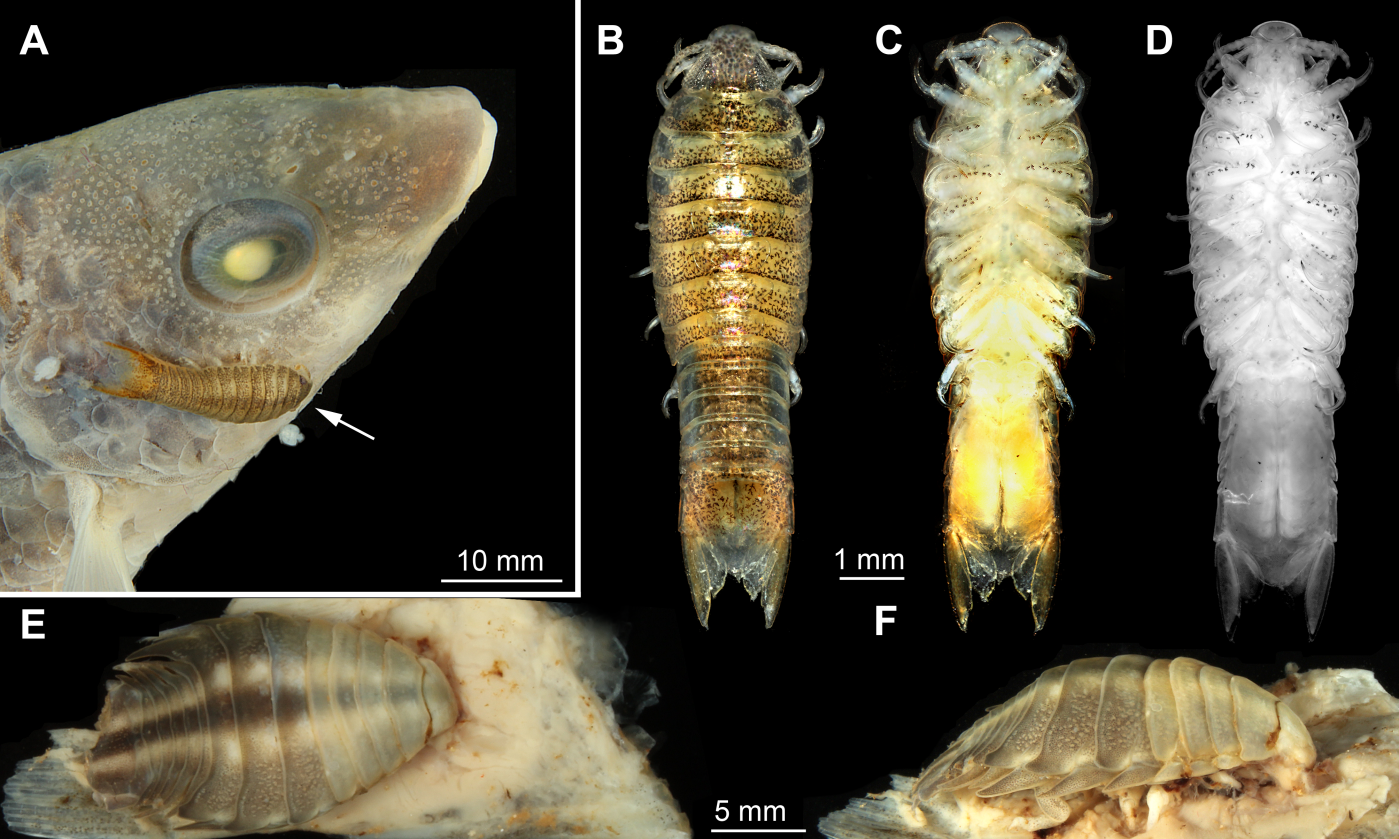
Macro- and fluorescence photography of the studied material. (A) dorso-lateral view of head of actinopterygid fish (*Symphodus cinereus*) with parasitic isopod (*Nerocila* sp.) specimen 1 attached to it. (B) dorsal view of specimen 1 (*Nerocila* sp.). (C) ventral view of specimen 1. (D) ventral view of specimen 1, fluorescence photography modified after Nagler et al. (2016). (E) dorsal view of specimen 2 (*Nerocila acuminata*) on isolated vertical fin of actinopterygid fish (Mugilidae). (F) lateral view of specimen 2 attached to vertical fin of a mugilid.

We investigated three parasitic isopod specimens. Specimen 1 (identified as *Nerocila sp*.): possible juvenile (9 mm), Cross Bay, Rovinji, Croatia (45°7.06′N 13°3.99′E), attached to *Symphodus cinereus* (Bonnaterre, 1788), Roland Melzer 2014 (ZSMA20159002) (Figs. 1A–1D).

Specimen 2 (identified as *Nerocila acuminata* Leach, 1818): ovigerous female (20 mm), Cross Bay, Rovinji, Croatia (45°7.06′N 13°3.99′E), attached to the vertical fin of a representative of Mugilidae Cuvier, 1829, Roland Melzer 2014 (ZSMA20159001) (Figs. 1E–1F).

Specimen 3 (identified as *Nerocila bivittata* Risso, 1816): ovigerous female (13 mm), Rovinji, Croatia, attached to *Crenilabrus quinquemaculus* Risso, 1827, unknown collector 1972 (ZSMO4con035).

Identification was performed by colleagues from the Zoological State Collection Munich (ZSM) based on the determination key of Brusca, Coelho & Taiti (2001).

### Documentation methods

Specimens were investigated with macro photography, composite auto fluorescence imaging and x-ray micro-CT scanning.

Macro-photography (Figs. 1A–1C and 1E–1F) (combined with composite imaging) were both performed (following e.g., Haug et al., 2011a; Haug et al., 2012; Haug et al., 2013) under cross-polarized light. We used a Canon EOS Rebel T3i camera, either with a Canon EFS (18–55 mm) lens (for overview images) or a Canon MP-E (65 mm) macro lens (for close-up images). Illumination was provided by a Canon Macro Twin Lite MT-24EX flash from the two opposing sides to provide even illumination.

**Figure 2:**
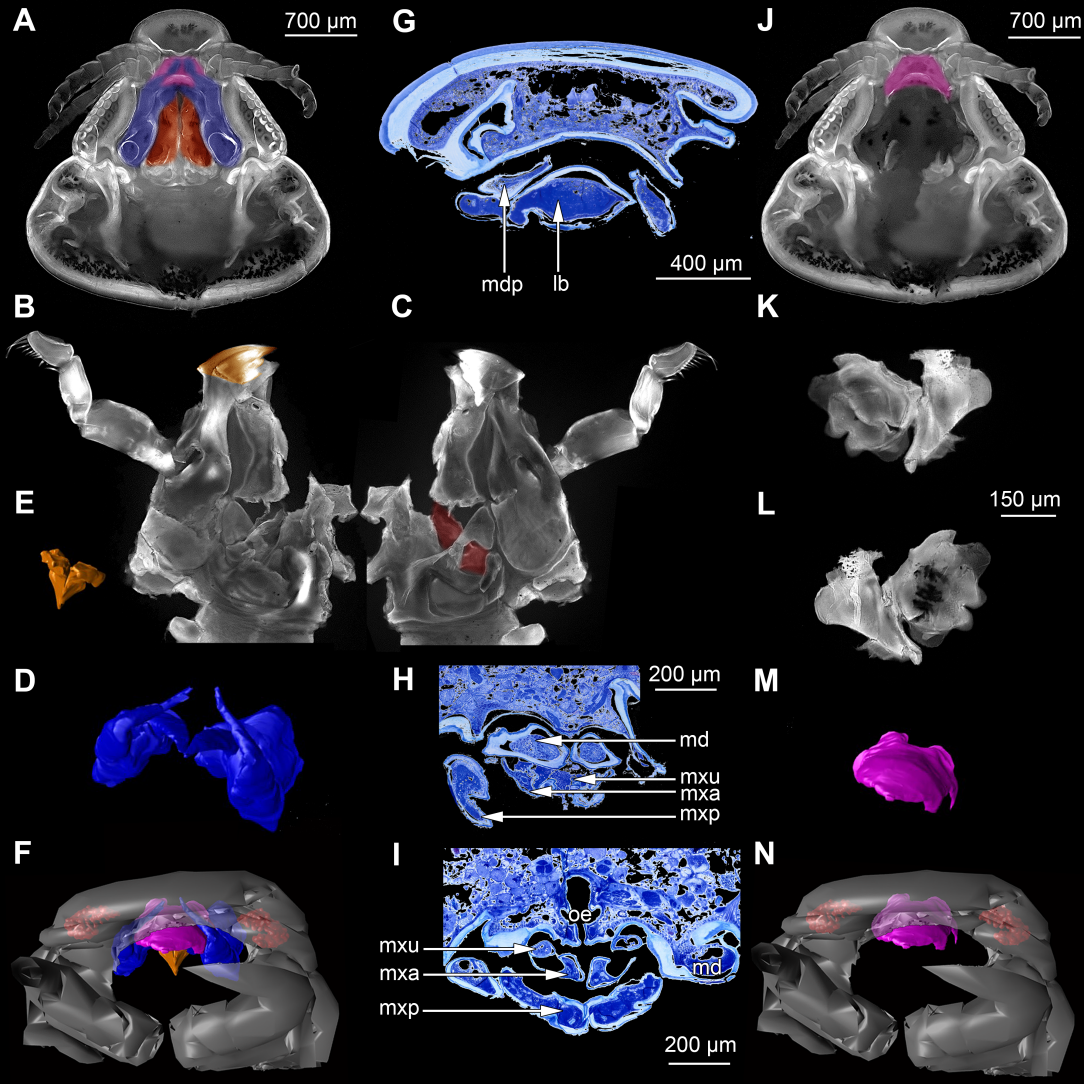
Fluorescence photography of mandibles and labrum of specimen 1 (A–C, J–L), respective surface models of specimen 2 (D–F, M–N) and histological sections of head of specimen 3 (*Nerocila bivittata*) (G–I). (A) ventral view of functional head of specimen 1, maxillipeds, maxilullae and maxillae removed to reveal anterior mouthparts. (B) anterior view of left mandible and paragnath of specimen 1, incisor region marked orange. (C) posterior view of left mandible and paragnath of specimen 1, leading channel for maxillula marked red. (D) surface model of mandibles of specimen 2. (E) surface model of paragnaths of specimen 2. (F) surface model of functional head of specimen 2 without maxillipeds, maxillae and maxillulae. (G) histological section of anterior third of head of specimen 3. (H) histological section through middle of head of specimen 3. (I) histological section of posterior part of head of specimen 3. (J) ventral view of functional head of specimen 1, maxillipeds, maxillae, maxillulae, paragnaths and mandibles removed to reveal anterior mouthparts. (K) posterior view of labrum of specimen 1. (L) anterior view of labrum of *Nerocila* sp. (M) surface model of labrum of specimen 2. (N) surface model of functional head of specimen 2 without maxillipeds, maxillae, maxillulae, paragnaths and mandibles. Surface models not to scale. Abbreviations: lb, labrum; mdp, mandibular palp; md, mandibel; mxu, maxillula (foremost spines); mxa, maxilla; mxp, maxilliped; oe, oesophagus.

**Figure 3:**
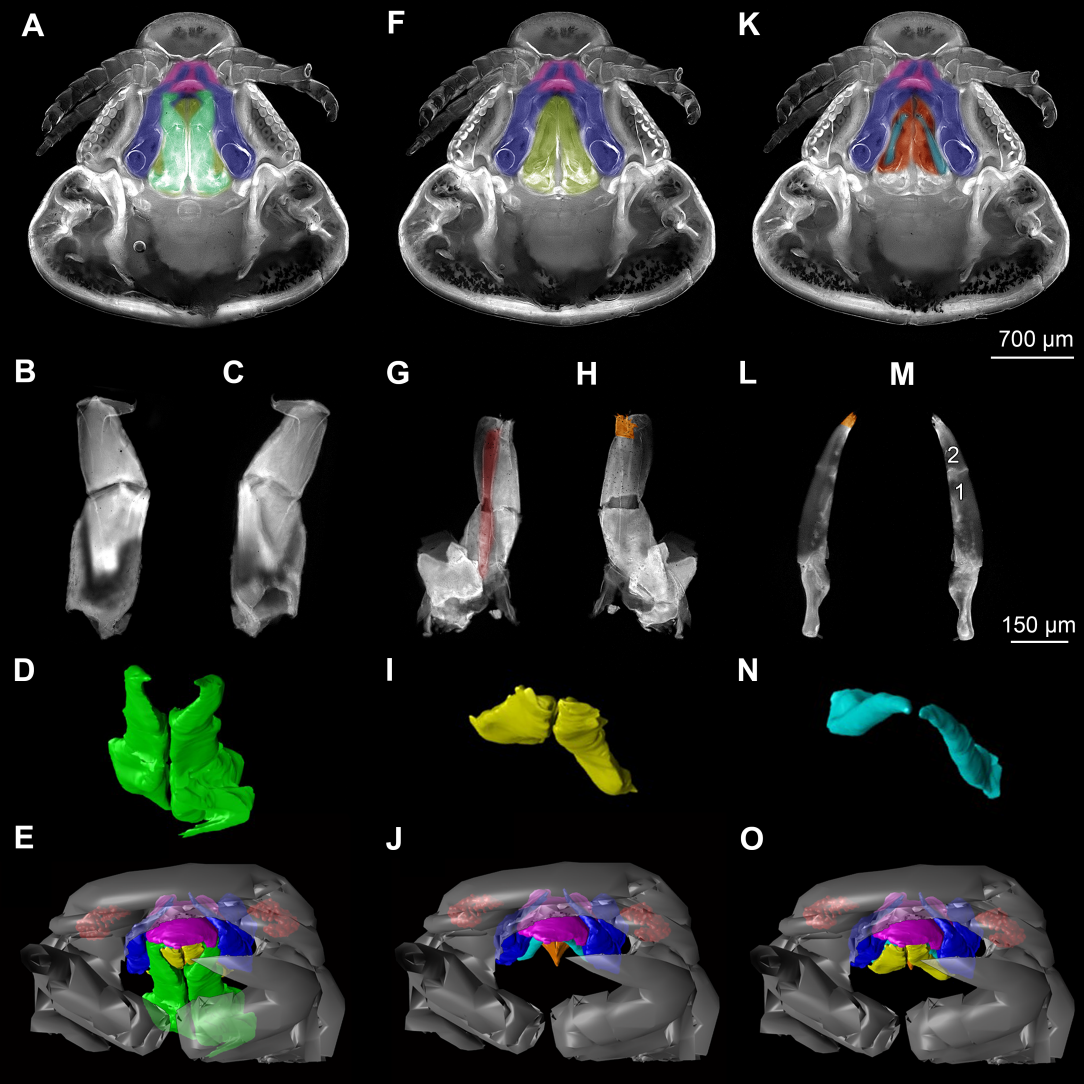
Fluorescence photography of mouthparts (maxillipeds, maxillae and maxillulae) of specimen 1 (*Nerocila* sp.) (A–C, F–H, K–M) and respective surface models of specimen 2 (*Nerocila acuminata*) (D–E, I–J, N–O). (A) ventral view of functional head of specimen 1. (B) posterior view of right maxilliped of specimen 1. (C) anterior view of right maxilliped of specimen 1. (D) surface model of maxillipeds of specimen 2. (E) surface model of functional head of specimen 2. (F) ventral view of functional head of specimen 1, maxillipeds removed to reveal anterior mouthparts. (G) anterior view of left maxilla of specimen 1, leading channel for maxillula marked red. (H) posterior view of left maxilla of specimen 1, spines marked in orange. (I) surface model of maxillae of specimen 2. (J) surface model of functional head of specimen 2 without maxillipeds. (K) ventral view of functional head of specimen 1, maxillipeds and maxillae removed to reveal anterior mouthparts. (L) posterior view of left maxillula of specimen 1, spines marked in orange. (M) anterior view of left maxillula of specimen 1. (N) surface model of maxillulae of specimen 2. (O) surface model of functional head of specimen 2 without maxillipeds and maxillae. Surface models not to scale.

Fluorescence microscopy (Figs. 1D, 2A–2C, 2J–2L, 3A–3C, 3F–3H and 3K–3M) of the functional head (cephalothorax) and isolated mouthparts after dissection of specimen 1 was performed on an inverse fluorescence microscope BZ-9000 (BIOREVO, Keyence) with a DAPI filter (*λ* = 358–461 nm) recording auto fluorescence and 4×, 10×and 20×lenses resulting in about 40, 100 and 200 magnification.

Stacks of images were processed with the freeware packages CombineZP (Alan Hadley), ImageAnalyzer (Meesoft) and ImageJ (Wayne Rasband). Assembling of stereo images and final processing (levels, sharpness, and saturation) was performed in Adobe Photoshop CS4.

**Figure 4:**
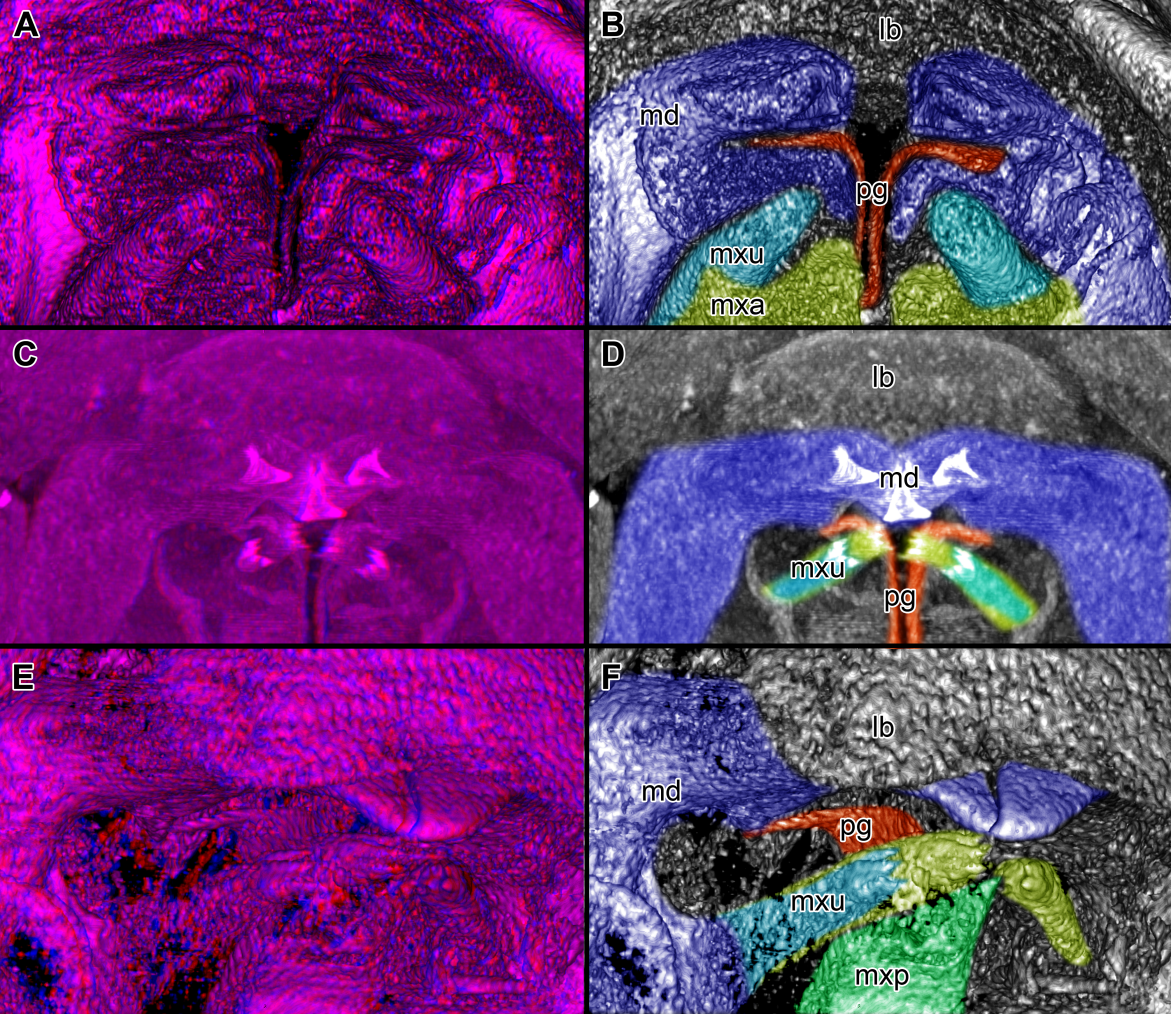
Stereo image of volume rendering of details of the mouthparts of specimen 2 (*Nerocila acuminata*). Please use stereo glasses for (A, C) and (E). (A) Stereo image of volume rendering of vertical section in posterior area of the mouthparts. (B) Color marked version of (A). (C) stereo image of volume rendering of ventral view of the mouth cone, the chitinous parts are highlighted. Note that the isopod is in a molting stage, one can see the new cuticle inside the mandibels and the maxillula in very bright color. (D) color marked version of (C). (E) stereo image of volume rendering of anterior-lateral view of the mouthparts, lb is only partly reconstructed. (F) color marked version of 4. Not to scale. Abbreviation: lb, labrum; md, mandibles; pg, paragnaths; mxu, maxillulae; mxa, maxillae; mxp, maxillipeds.

**Figure 5:**
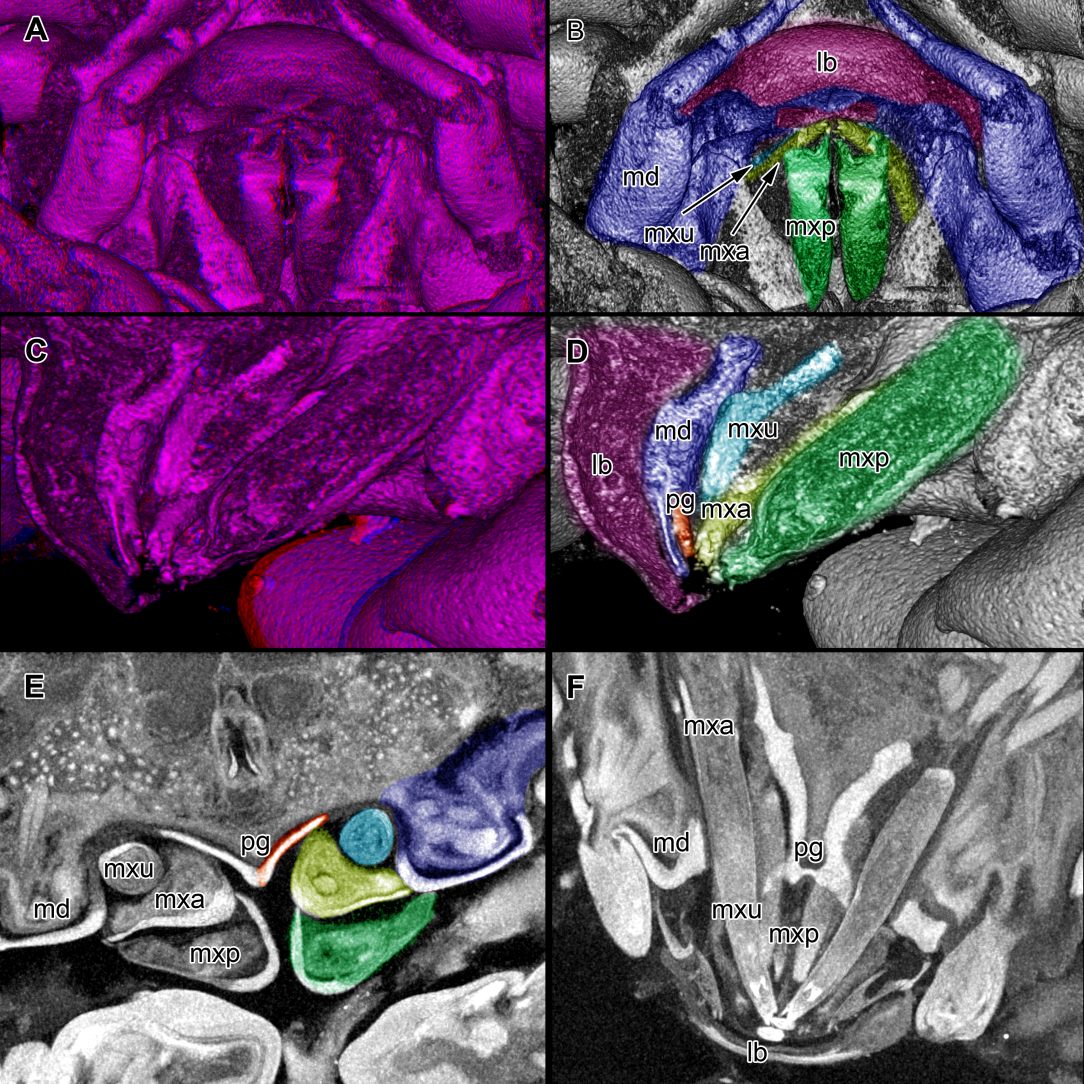
Stereo image of volume rendering (A, C) and virtual sections (B, D–F) through the mouthparts of specimen 2 (*Nerocila acuminata*). Note the overall cone-shape of the mouthparts together in (D, F). Please use stereo glasses for (A, C). (A) stereo model of ventral view of mouth cone. (B) color marked version of (A). (C) virtual, lateral stereo section through mouthparts. (D) color marked version of (C). (E) virtual, vertical section through mouthparts. (F) virtual, diagonal section through mouthparts. Not to scale. Abbreviations: lb, labrum; md, mandibles; pg, paragnaths; mxu, maxillulae; mxa, maxillae; mxp, maxillipeds.

**Figure 6:**
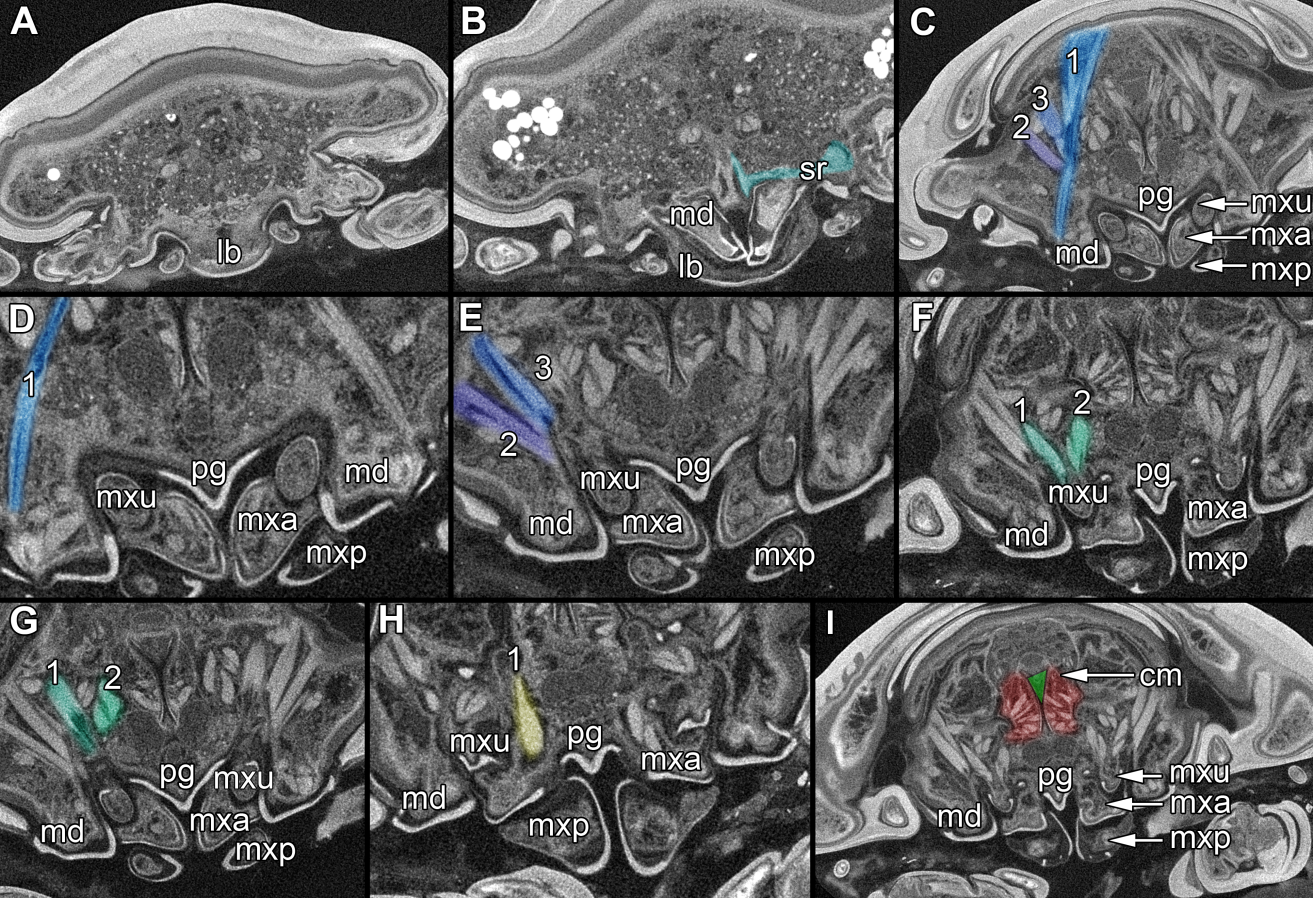
Single frames out of CT-Scan of functional head of specimen 2 (*Nerocila acuminata*). (A) functional head with labrum without muscles. (B) functional head with labrum, mandibles and a strong sclerotized region. (C) functional head with mandibles with three muscles 1–3, paragnaths, maxillulae, maxillae and maxillipeds. (D) functional head with mandibles and adductor (1). (E) functional head with mandibles and two abductors (2, 3), paragnaths, maxillulae, maxillae and maxillipeds. (F) functional head with mandibles, paragnaths, maxillulae with two muscles (1, 2), maxillae and maxillipeds. (G) functional head with mandibles, paragnaths, maxillulae with two muscles (1, 2), maxillae and maxillipeds. (H) functional head with mandibles, paragnaths, maxillulae, maxillae with one muscle (1) and maxillipeds. (I) functional head with mandibles, paragnaths, maxillulae, maxillae, maxillipeds, compressor muscles around the esophagus and the triangulate opening. Not to scale (lb, labrum; md, mandibles; pg, paragnaths; mxu, maxillulae; mxa, maxillae; mxp, maxillipeds; sr, sclerotized region; cm, compressor muscles.

Micro-CT scanning of specimen 2 was performed with a Nanotom M Phoenix (GE Sensing & Inspection Technologies GmbH). An overview scan ran 54 min with 100 kV and 100 µA, resulting in a calculated voxel size of 9 µm^3^. A scan of smaller details was performed with 90 kV and 110 µA, 54 min, resulting in a calculated voxel size of 2.3 µm^3^. Scans were reconstructed to tiff stacks with the build in software. Tiff stacks were further processed with ImageJ and Osirix 5.8.2 (Antoine Rosset). Surface models (Figs. 2D–2F, 2J–3L, 3D–3E, 3I–3J, 3N–3O, 4, 5 and 6) created (“segmented” or by thresholds) in Osirix were further modified and rendered with Blender 2.49 (Blender Foundation).

For histological studies (Figs. 2G–2I), the head of specimen 3 has been decalcified for 36 h in 5% pure acetic acid. After washing (three times for 2 h) in Sørensen phosphate-buffer (0.1 M) the specimen was transferred to 96% ethanol (via the following steps 10%, 30%, 40%, 50%, 70%, 80%, 90%, 96%, each for 1 day). After embedding in Historesin (Technovit 7100, Heraeus Kulzer GmbH) for 2 weeks, the polymerized block was sectioned with a microtome (820 rotary microtome, AO Spencer) in 2 µm slices from the anterior-ventrally side of the isopod’s head. Six sections in a row were transferred to object slides (Standard, Carl Roth) and dyed with Rydeberger solution (0.1 g methylene blue, 0.1 g thionin, 1.42 g disodium phosphate, 70 ml distilled water, 30 ml glycerin) for 30 s on a heating plate (70°C). After washing with distilled water, the slides were covered with a mounting media (DPX new, Merck) and a cover slide. They were dried for 72 h on a heating plate (40°C). The sections were scanned and digitalized with an Olympus-dotSlide (digital virtual microscope). All slides (124) were transferred to the Zoological State Collection Munich (ZSMO4con035).

### Presentation method

**Figure 7:**
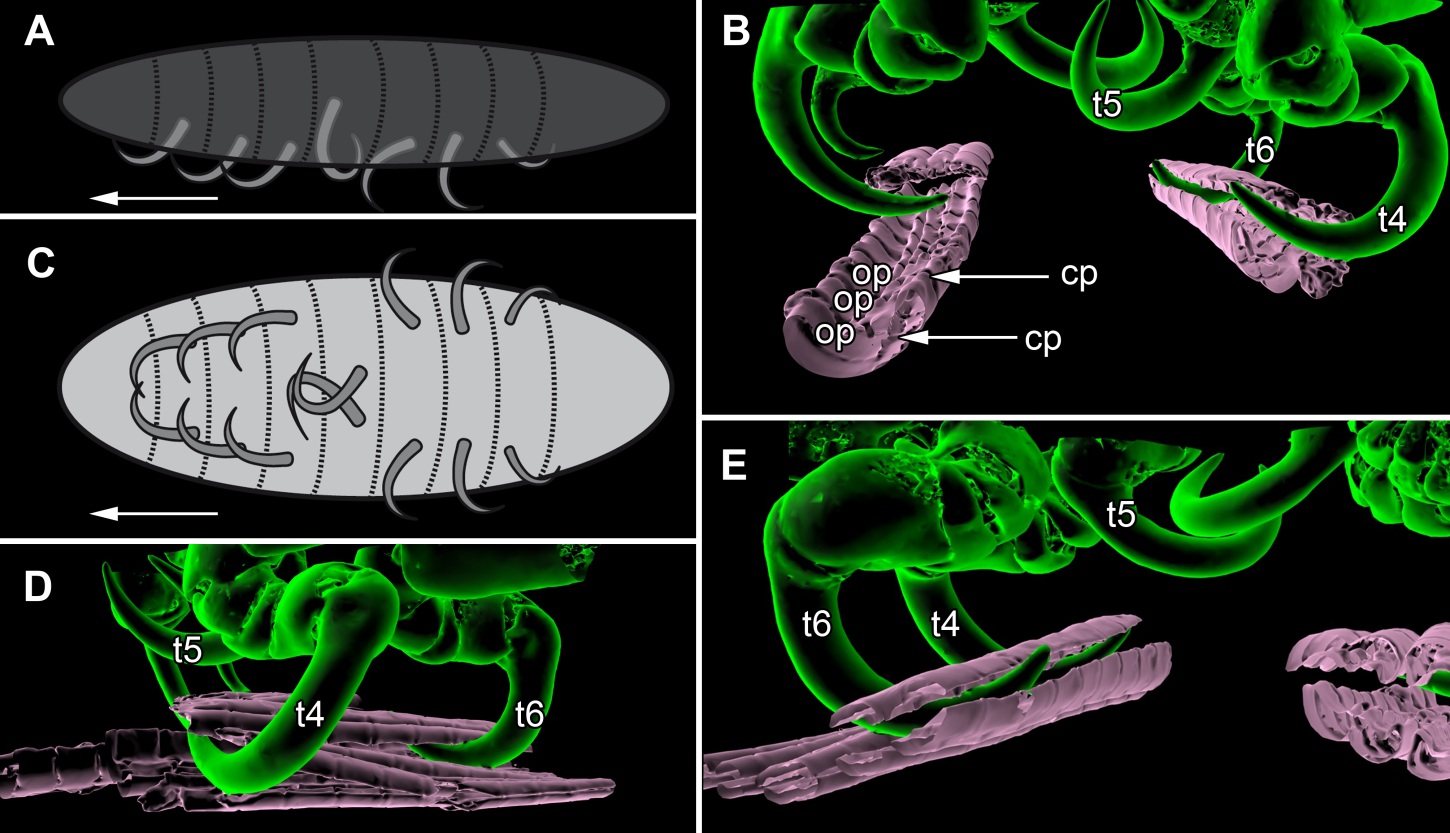
Details of the attachment with the thoracopods of specimen 2 (*Nerocila acuminata*). (A) diagram of orientation of thoracopods of specimen 1 in lateral view. (B) surface model of attachment of thoracopods IV–VI of specimen 2 to the cartaliginous parts between the ossified parts of the fin rays of vertical fin of actinopterygid fish (Mugilidae). (C) diagram of orientation of thoracopods of specimen 1 in ventral view. (D) lateral view of attachment of thoracopods IV–VI of specimen 2. (E) posterior view of attachment of thoracopods IV–VI of specimen 2. Not to scale. Abbreviations: cp, cartaliginous parts; op, ossified parts; t4–6, thoracopod IV–VI.

Description is focused on mouthparts and thoracopods, as these appendages are in direct contact with the host. For a better recognition we present color-marked images of the important appendages. For a better orientation we marked the eyes red (Figs. 2F, 2N, 3E, 3J and 3O). The labrum (lb) is marked purple (Figs. 2A, 2F, 2J, 2M–2N, 3A, 3E, 3F, 3J, 3K, 3O, 5B and 5D). The mandibles (md) are marked blue (Figs. 2A, 2D, 2F, 3A, 3E, 3F, 3J, 3K, 3O, 4B, 4D, 4F, 5B and 5D–5E). The paragnaths (pg) are marked brown (Figs. 2A, 2E–2F, 3J, 3K, 4B, 4D, 4F and 5D–5E). The maxillulae (mxu) are marked cyan (Figs. 3J, 3K, 3N–3O, 4B, 4D, 4F, 5B and 5D–5E). The (second) maxillae (mx) are marked yellow (Figs. 3A, 3E, 3B, 3I, 3O, 4B, 5B and 5D–5E). The maxillipeds (mxp) are marked green (Figs. 3A, 3D–3E, 4B, 4D, 4F, 5B and 5D–5E). The further posterior thoracopods (t) are marked neon-green (Fig. 7). The rays of the vertical fin (fr) of the examined specimen of Mugilidae are marked in pink (Figs. 7B and 7D–7E).

### Terminology

Simonetta & Delle Cave (1981) have expressed the necessity for a uniform terminology among arthropod workers. We absolutely agree on this notion. This does not mean we should force the use of specific terms; it merely means we should choose expressions that allow an unambiguous connection of term and structure. While one could think that this leads to more and more specialist terminology the opposite is true, specialist terminology tends to impede clear communication, as specialists in different groups use the same terms for different structures. This is the case for example for ‘pereiopod’ (or ‘peraeopod’). The ‘first pereiopod’ is in isopods the appendage of the 7th post-ocular segment, in decapods the appendage of the 9th post-ocular segment and in stomatopods the appendage of the 11th post-ocular segment. We therefore use thoracopod instead starting to count with the first appendage after the maxilla. We also avoid terminology implying serial homology of structures that have independent evolutionary origins, hence we use antennula and antenna (instead of antenna one and two) and maxillula and maxilla (instead of maxilla one and two).

Furthermore we need to point out our use of representatives of certain groups. It appears to be a common habit to use ‘cymothoid’ for a representative of Cymothoidae, but also for a representative of Cymothoida (which may be equivalent for some but not for all). To make a clear distinction between these two we use ‘cymothoiid’ as the correct derivation from Cymothoidae and ‘cymothoidan’ as a derivation from Cymothoida.

## Results

### Body organization

The body of the observed cymothoid isopods (Figs. 1B–1C) is organized into three major units:

(1) A functional head (cephalothorax) that consists of the eucrustacean head and the first thorax segment. The functional head bears the eyes, labrum, antennulae (“first antennae”), (“second”) antennae, mandibles, paragnaths, maxillulae (“first maxillae”), (“second”) maxillae and maxillipeds (thoracopods I).
(2) The posterior thorax (pereon) with seven segments, each bearing one pair of appendages, thoracopods II–VIII (pereopods I–VII). All tergites (dorsal sclerotisations) are extended at the latero-distal ends. From the anterior towards the posterior part of the thorax, these extensions increase in size.
(3) Pleon with five free pleonal segments and the pleotelson that consists of the sixth pleonal segment and the telson. The tergites of the pleomeres have distal extensions similar to those on the thoracic tergites. These become smaller and thinner towards the posterior end. The pleotelson forms a broad rounded plate.

### Structures of the functional head

#### Eye:

The compound eyes (e) are shaped like a broad three-dimensional sickle and consist of 26 single ommatidia each (Fig. 2A). They are located antero-laterally and dorsally inside the cephalothorax, each on the lateral side next to the mouthparts (mandibles, maxillulae, maxillae, paragnaths).

#### Antennula and antenna:

Antennula with eight elements each. Antenna with nine elements each (Fig. 2A).

#### Labrum:

Labrum anteriorly arising from a sclerotised plate, the clypeus. Labrum (lb) forms a broad, arched rounded triangle with a tip on the front and covers all other mouthparts from anterior. It is crescent-shaped (distal view) curved. The central aspect is covered by a thick cuticle as opposed to the thinner cuticle on the lateral aspect (Figs. 2K–2N and 2G). No musculature was observed within the labrum (Fig. 6A), indicating that this structure is not actively movable.

#### Mandible:

Mandible (md) with prominent proximal part (coxa) and elongated distal part with three elements (palp) (Figs. 2A–2D and 2G–2I). Coxa large and stout. The proximal region is prolonged into a lobe-like endite that partly surrounds the paragnaths (see also below) (Figs. 4A–4B). Disto-medially the coxa is drawn out or elongated into a blade-like process with a massive edge (incisor) at the distal end.

The incisor of both mandibles is strongly sclerotized. The two incisors are situated opposing each other and are directed disto-medially (instead of medially) (Figs. 4E–4F, 5A–5B). The blade-like process is connected via one muscle bundle to a tendon that is adhered to a strongly sclerotized region (Fig. 6B) between the head capsule and the mandible.

On each mandible there are three relatively large muscle bundles, filling most of the lumen (Fig. 6C):

(1) an adductor from the inside of the proximal part of the mandible to the inner side of the head capsule, where it is broadly adherent (Fig. 6D)
(2) a first abductor from the proximal lateral side of the mandible to the lateral inner side of the head capsule (Fig. 6E)
(3) a second abductor from a bit more dorsal than the first of the proximal lateral side of the mandible to the proximal lateral inner side of the head capsule (Fig. 6E).

The long and relatively large palp of each mandible consists of three elements. The distal one bears several setae and spines that increase towards the distal end. There is one muscle bundle from the proximal element to the insertion point of the mandible palp on the coxa. They ‘grasp’ around the labrum from the latero-anterior side (Figs. 2B–2E, 2G and 5A–5B).

#### Paragnath:

Paragnath, hypopharynx or lower lip (pg) (Figs. 2A–2C and 2E–2F) as a protuberance of the sternum forms a backward pointing triangle (in ventral view), comparable to a funnel in the ventral midline.

Anteriorly (Figs. 4A–4F and 5F) the edges of the paragnaths form on each side a rail for the mandible (in fact it was not possible to separate mandible from paragnath, also due to the lobe-like endite of mandible). Posteriorly the edges form on each side together with the lobe-like endite of the mandible a rail for maxillula (Fig. 5E). No apparent joints of the paragnath could be observed, indicating that they are attached and not movable. However, they could be indirectly movable through muscle bundles attached to their bases. Their midway part is strongly sclerotized (Figs. 6C–6I).

#### Maxillula:

Maxillula (mxu) is slender and elongated with two elements (Figs. 3K–3O). The distal end of each is sharp with four recurved pointed spines (Figs. 2H and 3L). The maxillula is protruding further distally than any other mouthpart. The maxillula is guided by the paragnath and mandible anteriorly and by the maxilla posteriorly (Figs. 2I and 5E–5F).

One muscle that is formed by many single muscle strands is located at the proximal inner side of the second element of the maxillula and at the contrary inner side of the head capsule. The retractor muscle is located in the outer side of second element of maxillula and at the inner side of the head capsule (Figs. 6F–6G). Between the individual elements of the maxillula there are several small muscle bundles, connected to the proximal inner side of the first element and the antero-median side of the second element.

#### Maxilla:

Maxilla (mxa) is elongated, chisel-like and flattened in anterior–posterior axis (Figs. 2H and 3F–3J). It bears ventrally posterior directed hooked spines and forms dorsally a rail-like groove for the maxillula (Figs. 2I and 3G–3H). One muscle connects the maxilla to the proximal edge to the head capsule (Fig. 6H). Further distally within the maxilla there are two muscles, connected to the middle and lateral lobes.

#### Maxilliped:

Maxilliped (mxp) is massive and stout with three elements (Figs. 2I and 3A–3E). The distal one is modified into a prominent hook-like claw with a sharp, thin tip. This claw-like element grasps around the other mouthparts, except the labrum and the mandibular palps, laterally (Figs. 5A–5B). There are two crossing muscles inside the distal element of maxilliped.

#### Overall arrangement:

Overall the mouthparts together form a short and broad truncated cone. Inside this cone they additionally form a tube, which is anteriorly confined by the labrum, posteriorly by the maxillipeds and laterally by the paragnaths and maxillae. The distal ends of the maxillulae and the incisor region of the mandibles can protrude from the cone, when moving.

The oral opening forms a typical Y (Fig. 2I), an ideal shape for sucking, and is surrounded by a strong compressor muscle (Figs. 2I and 6I).

### Appendages on the thorax

All thoracopods (II–VIII) consist of seven elements (coxa, basis, ischium, merus, carpus, propodus, dactylus). The dactylus is longer than the propodus on each thoracopod. It is strongly curved and elongated to a pointed spine-like hook. When attaching to the fin, the attachment of the dactyli is always within the cartilaginous parts between the ossified parts of the fin rays of the host fish (Figs. 7B, 7D and 7E).

#### Thoracopod II–thoracopod IV:

Their dactyli are curved to the ventro median of the isopods’ body. Thus the dactylus grasps into the host in a manner that the dactylus is directed at a 90°angle to the isopods‘ body.

#### Thoracopod V:

The dactylus is 20% longer than the dactylus of the other thoracopods. It is located closer to the median axis of the isopod andmuch more curved.

#### Thoracopod VI and VII:

The merus, the carpus and the propodus are directed diagonally towards the posterior outer edge of the isopod. The dactylus grasps into the host in a way that the dactylus lays more inclined backwards.

#### Thoracopod VIII:

The merus, the carpus and the propodus are directed to the posterior part of the isopod. The dactylus of thoracopod VII is 20% smaller than the dactylus of thoracopod II–IV, VI–VII. After attachment it lays in an almost parallel position to the isopod’s body. Only the distal part of the dactylus grasps into the host (Figs. 7A and 7C).

## Discussion

### Choice of specimens

The number of studied specimens is comparably low and not all belong to the same species of *Nerocila*. Also, species identification is in fact difficult, especially concerning relatively young specimens. Yet unlike a taxonomic study, this is not of importance for the questions being raised here. All specimens have been identified as representatives of *Nerocila*. This group is generally resolved as the sister group to all remaining cymothoiids and generally assumed to represent a rather plesiomorphic morphology. It has been suggested that cymothoiids evolved from a *Nerocila*-like ancestor (Brusca, 1981). With this, it is the perfect group to start with. Larger amounts of specimens were not available for this study and it would have been challenging to study more specimens to the same detailed degree as presented here.

The aim of this study was to understand the basic morphological interactions of the mouthparts and their adaptations. Hence, interspecific variations or even intraspecific variations are not of interest.

Comparing our results to the earlier study by Günther (1931), we can largely support his observations. The main advantage of the ‘modern-day’ methods applied is the possibility to look inside the still interconnected mouthparts. Below we will try to infer functional aspects based on the observed morphological details.

### Sucking mouthparts

Sucking mouthparts are widespread among various groups of parasitic arthropods (e.g., Bergman, 1996; Blanke et al., 2015). As previous studies strongly suggest cymothoiids are adapted to parasitism (Brusca, 1981; Brusca & Iverson, 1985; Brusca & Wilson, 1991; Brandt & Poore, 2003); the question arises in how far the mouthparts of the parasitic isopods described herein represents sucking mouthparts.

Günther (1931) has suggested that the cymothoiid mouthparts represent true sucking-type mouthparts. He also described the foregut (usually developed as a gizzard) functioning as a pump maw (comparable to that in spiders). This would only work if the mouthparts indeed form true suction mouthparts.

We can clearly show that the mouthparts are tightly arranged together. The entire apparatus when virtually cut sagitally (Figs. 5C–5D) and (locally) horizontally (which is almost a cross-section; Figs. 5E–5F) reveals a triangular section with a distal tip. The overall arrangement is therefore that of a cone, hence a mouth cone. This is similar in other parasitic arthropods, yet the mouth cone in our specimens appears rather stout compared to that in other arthropods (Møller et al., 2008; Møller, 2009; Schwabe, Holtheuer & Schories, 2014). In an extreme case also the elongate beak-like extensions in mosquitoes and hemipterans could be seen as principal mouth cones (Labandeira, 1997). This basic arrangement of the mouthparts in the specimens investigated herein gives us a first indication that the mouth cone is indeed involved in sucking.

### The “outer seal” of the mouth cone: labrum, distal mandible, maxilliped

Brusca (1981) proposed that the labrum prevents a loss of liquid nutrients. This is plausible, given its observed morphology and position, partly covering most of the other mouthparts and thus sealing the mouth cone from anterior.

Based on its position, reaching around the labrum, the mandibular palp seems to “attach” to the labrum from anterior and further cramp the parts of the mouth cone together. However, due to the several strong setae (Figs. 2B–2C) they could be used for cleaning as known from other arthropods (Roberts, 1968; Garm, 2004; Møller, Olesen & Waloszek, 2007) and for additional attachment to the host during the feeding process. The paragnaths seem to serve the purpose of preventing food particles from being lost ventrally or laterally.

The maxillipeds act as a seal posteriorly. As their distal part is concealed by the labrum, the rigidity of the mouth cone is further stabilized. In the studied specimen, we could not observe spines on the terminal article, as observed for *Nerocila orbignyi* (Thatcher, 1997). The specimens studied herein seem not to perform ripping on the body surface of the host as suggested by Thatcher (1997).

The lack of proximal musculature within labrum and maxilliped indicate that these structures could not be moved in relation to the body. This indicates that the tight mouth cone arrangement provides a permanent “outer seal” for the mouthparts. Such a seal would be necessary for true sucking mouthparts.

### The structure of the inner cone: proximal mandible, paragnaths, maxillula, maxilla

Compared to non-parasitic mouthparts, the mouthparts of the specimens studied herein are specialized in two aspects:

(1) Orientation of the mouthparts. The “normal” orientation of mouthparts is along the lateral-median axis. In this way the opposing mouthparts can act against each other. In the case here all structures are rotated of this axis by at least 45 degrees distally, i.e., towards the host. This is not unusual in parasites and especially not in sucking mouthparts (Merrit et al., 1996; Ma et al., 2013).
(2) Mandible, paragnath, maxillula and maxilla are tightly ‘folded’ around each other. This provides guidance during movements, more precisely a restriction. This is coupled to the orientation, as the ‘normal’ arrangement of mouthparts allows an inwards movement (medially) but here the mouthparts need to move more distally. Hence, the different structures provide functional rails (guidance) for allowing such movements.

The paragnaths provide a guide for the mandibles on their anterior side. This arrangement is in principle not unusual and is wide spread within Eucrustacea (e.g., Waloszek, 1993; Waloszek et al., 2007; Haug et al., 2011b; Rötzer & Haug, 2015). Yet, here the case seems further specialized as not only the paragnath partly ‘grip’ around the mandible edge (Figs. 4C–4F; as also e.g., in mystacocarids; Haug et al., 2011b their Figs. 11A–11D and 16), but the mandible additionally possesses a specific protrusion gripping proximally around the paragnaths (Figs. 4A and 4B). Thus the paragnaths are ‘held’ by the mandibles in contrast to lying against the inner margin of them (as already observed by Brusca, 1981).

Maxillulae are surrounded by the endites of mandibles antero-distally, by the paragnaths antero-medially and by a ‘channel’ formed of mandibles, paragnaths and maxillae proximally. Overall the maxillae appear to be strongly modified as a rail for the maxillulae. The overall arrangement (including paragnaths and mandibular protrusion) could be compared to a cylinder in which the maxillula could be moved like a piston. For additional stability maxillae may insert their sharp spines at the distal end in the tissue of the host (Brusca, 1981) and press the plump, soft ends behind the spine to the surface of the host. Thus the attacking point of maxillulae cannot be shifted, as the distal part of maxillae is not independently moveable. Due to the location of the muscles, a movement in dorso-ventral direction is facilitated and the sealing of the mouthpart cone to the lateral is given by pressing maxillae towards the head and towards itself.

Overall the entire arrangement is quite tight and partly folded into each other. Folding of mouthparts is known in sucking mouthparts of insects (e.g., Krenn & Aspöck, 2012). This provides a further indication for true sucking mouthparts in our specimens.

Additional support is provided by the inner most part of the cone. Due to the tight arrangement of the mouthparts, the innermost part of the mouth cone is a rather narrow channel with y-shaped or tri-radiate cross sections. Such a narrow and confined arrangement would provide the necessary stability for a true suction tube. Together with the seal provided by the outer mouth cone, the channel indeed most likely represents a true suction tube, allowing transportation of liquids effectively without major loss.

### Penetrating the host surface: mandible and maxillula

As pointed out, the movements of the mouthparts are strongly confined. Still the presence of musculature indicates that (at least some) mouthparts are actively movable. Due to the (most likely permanent) seal of the outer cone the moving mouthparts, endite of the mandible and maxillula can only protrude distally from the mouth cone and only to a small degree.

The incisor region of the mandibles is reduced to a triangular blade-like process that should be able to injure the epidermis of the host (Brusca, 1981; Thatcher, 1997). Other hypotheses also have been proposed including shredding and masticating food, tightly gripping the host fish, handling the host (Al-Zahaby, El-Aal & El-Bar, 2001) or only piercing in the host‘s surface to penetrate blood vessels (Jithendran, Natarajan & Azad, 2008). It has been suggested that isopods also use the mandibles for cutting pieces of the host and crush them between the gnathal edges (Thatcher, 1997).

The location and morphology of the muscle bundles of the mandibles allow an inward–outward movement and an anterior–posterior movement possibly resulting in a slight rotation movement of the incisor region. For such a movement, a counter pressure is needed for not pushing themselves off the host. This pressure is produced by the attachment of the thoracopods (Günther, 1931) that press the isopod against the host (see also further below).

Although the range of movement of the mandibles is relatively small, it seems to be able to cut off small pieces from the host. This is further facilitated by the very sharp-appearing most distal region of the incisor regions. Thus, the spines on the edge can perform a type of grinding move against each other and cut the epidermis of the host.

### A mouth cone with a suction tube

Summing up, all mouthparts are strongly modified for a parasitic lifestyle (supporting earlier interpretations, e.g., Brusca, 1981; Brusca & Iverson, 1985; Brandt & Poore, 2003). The mouthparts are arranged in a tight well-sealed cone, which is interpreted to have a functional suction tube in the center.

With this, the host is injured on a very specific and relatively small region. This limits the lesion on the host and most likely provides the possibility for a longer time span for absorbing liquids from the host. To use this sucking tube in the most effective way, cymothoiids have to press their mouth cone strongly against the host fish.

Studies of the gut content of Cymothoidae have confirmed that host blood, but also pieces of muscle tissue (small enough to pass the suction tube) are the main component of cymothoiid diet (Romestand & Trilles, 1977; Bunkley-Williams & Williams Jr, 1998; Alas et al., 2008; Sethi, 2012; Al-Zubaidy & Mhaisen, 2013). This is in concordance with the observed morphology, and hence further supports the hypothesis that the mouthparts of cymothoiids are true sucking mouthparts.

### Attachment with the thoracopods

All representatives of *Nerocila* are ectoparasitic and attach on the side or the fins of numerous marine fishes. In the Mediterranean Sea these isopods are attached mostly to species of Labridae (Trilles, 1994; Charfi-Cheikhrouha et al., 2000) or Mugilidae (Öktener & Trilles, 2004; Ramdane, Bensouliah & Trilles, 2007).

Certainly cymothoiids use their thoracopods with their prominent dactyli to hold themselves onto the host (Brusca & Wilson, 1991; Smit, Bruce & Hadfield, 2014). Due to the deep insertion of the dactyli in the host tissue, some representatives of *Nerocila* can cause deep lesions with their thoracopods (Ghani, 2003).

As Brusca (1981) mentioned, the posterior thoracopods (thoracopod VI–VIII) are flexed against themselves, but not all thoracopods are oriented in the same way (Figs. 7A and 7C). Thus, the dactyli of thoracopod VI–VIII grasp in the host from a different angle in contrast to a 90°angle to the isopod length axis from thoracopods II–IV. Thoracopod V is in an even more median direction.

This type of arrangement could facilitate an even tighter attachment to the host and makes cymothoiids so difficult to remove. Additionally, this differentiation may also be coupled to the specific molting strategy in isopods. They molt in a biphasic manor (George, 1972).

First, they molt the pleotelson and pleon together with the posterior three thorax segments while the anterior part of the isopod (functional head + thoraxsegments II–V) stay anchored on the substrate, e.g., host or bottom (George, 1972; Segal, 1987). Then, the anterior part is molted after the posterior part has re-attached.

Representatives of Cymothoidae are known to stay permanently at the same position on their host after the first permanent attachment as a juvenile (Bunkley-Williams & Williams Jr, 1998; Fogelman, Kuris & Grutter, 2009). Most host fishes try to get rid of isopod parasites by rubbing against the bottom (Segal, 1987). As molting is known as a vulnerable stage in crustaceans (Steel, 1980; Sorach et al., 2013; Nguyen et al., 2014), during molting many isopods were dropped off their hosts. With their biphasic molting, the parasitic isopods can stay in contact with their host and after the molting they can re-attach completely again. The biphasic molting of isopods could therefore potentially be seen as a perfect pre-adaptation to this type of parasitic lifestyle.

### Further adaptations to parasitism

Besides the adaptations of the mouthparts and the thoracopods to a parasitic lifestyle, representatives of Cymothoidae show also further adaptations for this specific niche that they are fulfilling. In contrast to free-living, juvenile cymothoiids, obligatory parasitic adults have reduced visual capabilities (Brusca, 1981). Also, antennulae and antennae are reduced (Brusca & Wilson, 1991; Smit, Bruce & Hadfield, 2014) and their body is more dorso-ventrally flattened (Brusca & Iverson, 1985) reducing water resistance. A further reduction of drag is achieved by tapering posterior and anterior ends (Smit, Bruce & Hadfield, 2014).

Due to this streamlined body shape, the fishes do not lose much more energy than without parasites. This is important for fishes to escape larger predators (Fogelman, Kuris & Grutter, 2009) and also makes it more difficult for the host, or cleaner fish to remove the parasite. With respect to cleaner fish, an adaptation of the pigmentation to the surrounding tissue has also been reported (Grutter, 2002).

During the ontogenetic process towards adulthood, parasitic isopods lose their swimming ability and use their pleopods only for respiration (Brusca & Wilson, 1991). Also, the overall body shape is influenced by the attachment to the host: often cymothoiids are twisted to one side in response to the growing on the host (Brusca & Iverson, 1985; Smit, Bruce & Hadfield, 2014), e.g., growing around the fin (Brusca, 1981; Nagler et al., 2016). Overall, while the studied cymothoiids still pose an easy to recognize isopod shape compared to other parasitic isopods, e.g., Dajiidae, Bopyridae, they seem to possess numerous adaptations to their parasitic lifestyle.

### Adaptations of arthropods to parasitic lifestyles

Al-Zahaby, El-Aal & El-Bar (2001, p. 25) suggested that insects have ‘more potentially developed and highly modified’ mouthparts than crustaceans. This may not be correct. First, insects are an ingroup of Crustacea sensu lato (e.g., Wolff & Scholtz, 2006; Haug, Maas & Waloszek, 2010). Second, among eucrustacean ingroups there are forms that are so heavily modified that it is almost impossible to recognize them as arthropods in their adult form, such as parasitic representatives of Copepoda, Facetotecta, Rhizocephala or Bopyridae (e.g., Rohde, 2005; Klompmaker & Boxshall, 2015). Here only the larval stages still show that these forms are indeed ingroup eucrustaceans. Even strongly modified insect parasites/parasitoids such as strepsipterans do not reach such a degree of “dedifferentiation” (Kathirithamby, 2009; Nagler & Haug, 2015). This means that the mentioned non-insect crustacean group reduce their overall body organization concerning inner and outer segmentation and/or specialization of appendages, or reduce the latter to a large degree at all.

Hence, as pointed out above, there is no reason to indicate that cymothoiids are less drastically adapted to their parasitic lifestyle than insects. In fact, concerning their mouthpart adaptations, some insect ingroups may provide the closest comparisons.

## Conclusion and Outlook

Although we investigate only three specimens, we show that cymothoiid isopods possess numerous specializations coupled to their parasitic lifestyle. Most important are:

(1) Mouthparts that form a tightly sealed mouth cone with a central suction tube.
(2) Thoracopods that provide a tight attachment to the host fish, coupled with the biphasic molting style providing a permanent attachment to the host.
  For a better understanding of how such parasitism can evolve these detailed data together with fossil specimens are necessary. Comparing fossil specimens with the results of this study, we will be able to identify also possible parasites among disarticulated fossil isopods as we can expect certain characters as described for their modern relatives, e.g., mouthparts arranged in a cone, mandible and maxillula tilted off axis, thoracopods II–VIII with hook-like dactyli. These will then allow a more detailed reconstruction of character evolution towards the modern-type fish parasites.

## Supplemental Information

10.7717/peerj.2188/supp-1 Supplemental Information 1 Fig 4A Raw

10.7717/peerj.2188/supp-2 Supplemental Information 2 Fig 4C Raw

10.7717/peerj.2188/supp-3 Supplemental Information 3 Fig 4E Raw

10.7717/peerj.2188/supp-4 Supplemental Information 4 Fig 5A Raw

10.7717/peerj.2188/supp-5 Supplemental Information 5 Fig 5C Raw

10.7717/peerj.2188/supp-6 Supplemental Information 6 Fig 5E Raw

10.7717/peerj.2188/supp-7 Supplemental Information 7 Fig 6A Raw

10.7717/peerj.2188/supp-8 Supplemental Information 8 Fig 6B Raw

10.7717/peerj.2188/supp-9 Supplemental Information 9 Fig 6C Raw

10.7717/peerj.2188/supp-10 Supplemental Information 10 Fig 6D Raw

10.7717/peerj.2188/supp-11 Supplemental Information 11 Fig 6E Raw

10.7717/peerj.2188/supp-12 Supplemental Information 12 Fig 6F Raw

10.7717/peerj.2188/supp-13 Supplemental Information 13 Fig 6G Raw

10.7717/peerj.2188/supp-14 Supplemental Information 14 Fig 6H Raw

10.7717/peerj.2188/supp-15 Supplemental Information 15 Fig 6I Raw

## Additional Information and Declarations

### Data Availability

The following information was supplied regarding data availability: FigShare: *Nerocila acuminata*: https://figshare.com/s/6684911ebe4f4fe02cf0
*Nerocila* mouthparts: https://figshare.com/s/76d87aba6fc81de6c31e.
