# Supplementary figures and images for: Functional morphology of parasitic isopods: understanding morphological adaptations of attachment and feeding structures in Nerocila as a pre-requisite for reconstructing the evolution of Cymothoidae
Source: PeerJ. 2016 Jul 5;4:e2188. doi: 10.7717/peerj.2188 (PMC4941765; doi:10.7717/peerj.2188)

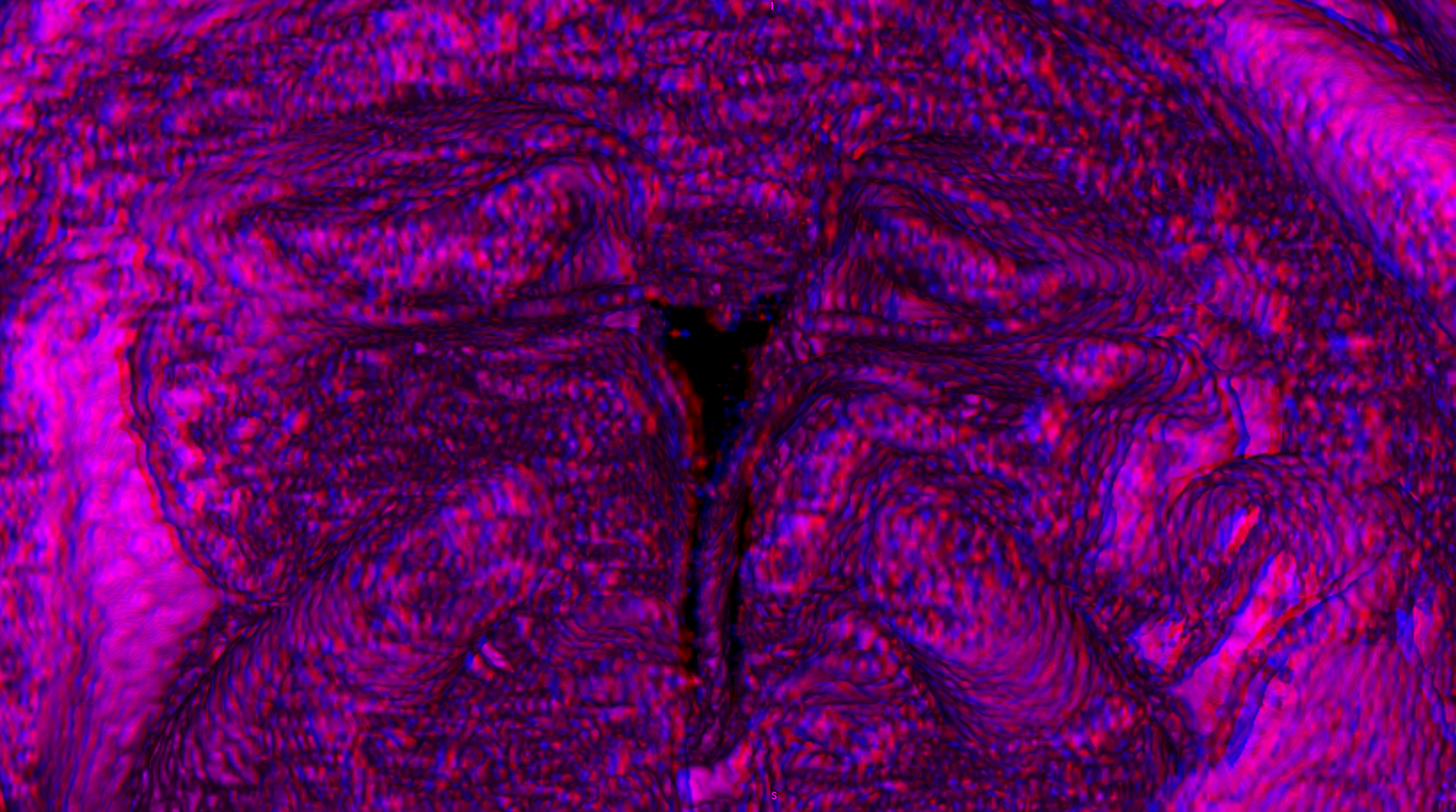

Supplement: Supplemental Information 1 [file peerj-04-2188-s001.png]

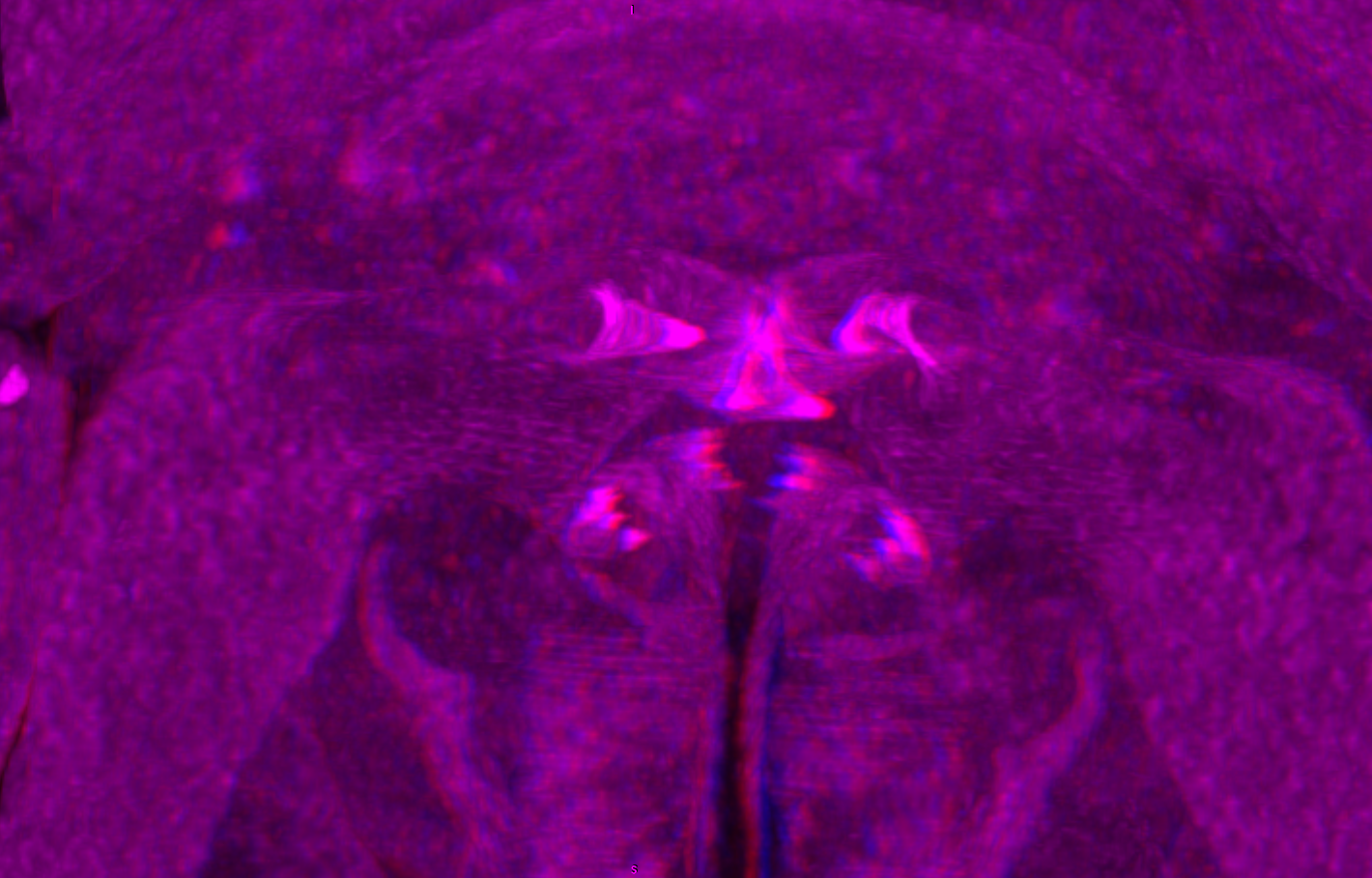

Supplement: Supplemental Information 2 [file peerj-04-2188-s002.png]

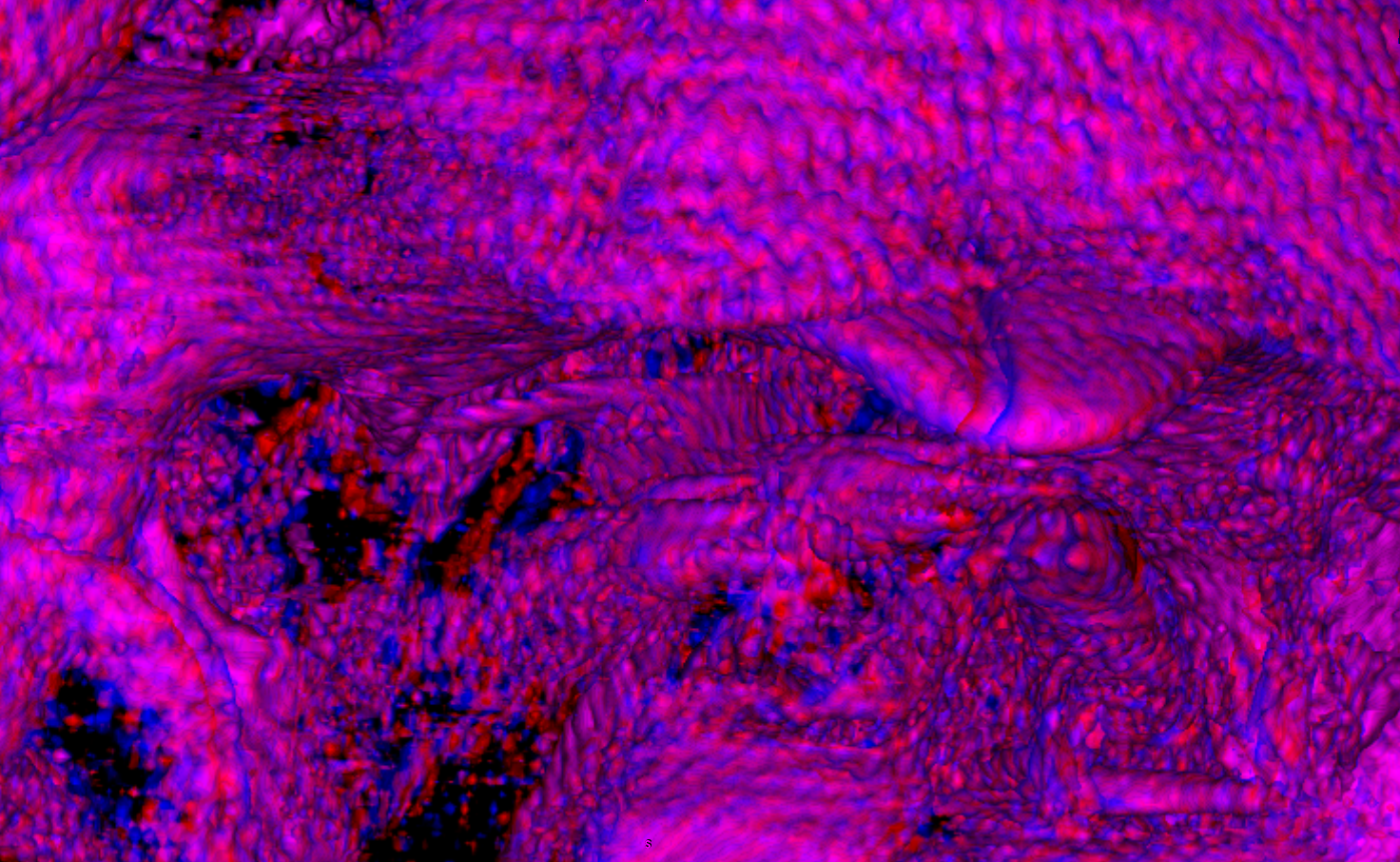

Supplement: Supplemental Information 3 [file peerj-04-2188-s003.png]

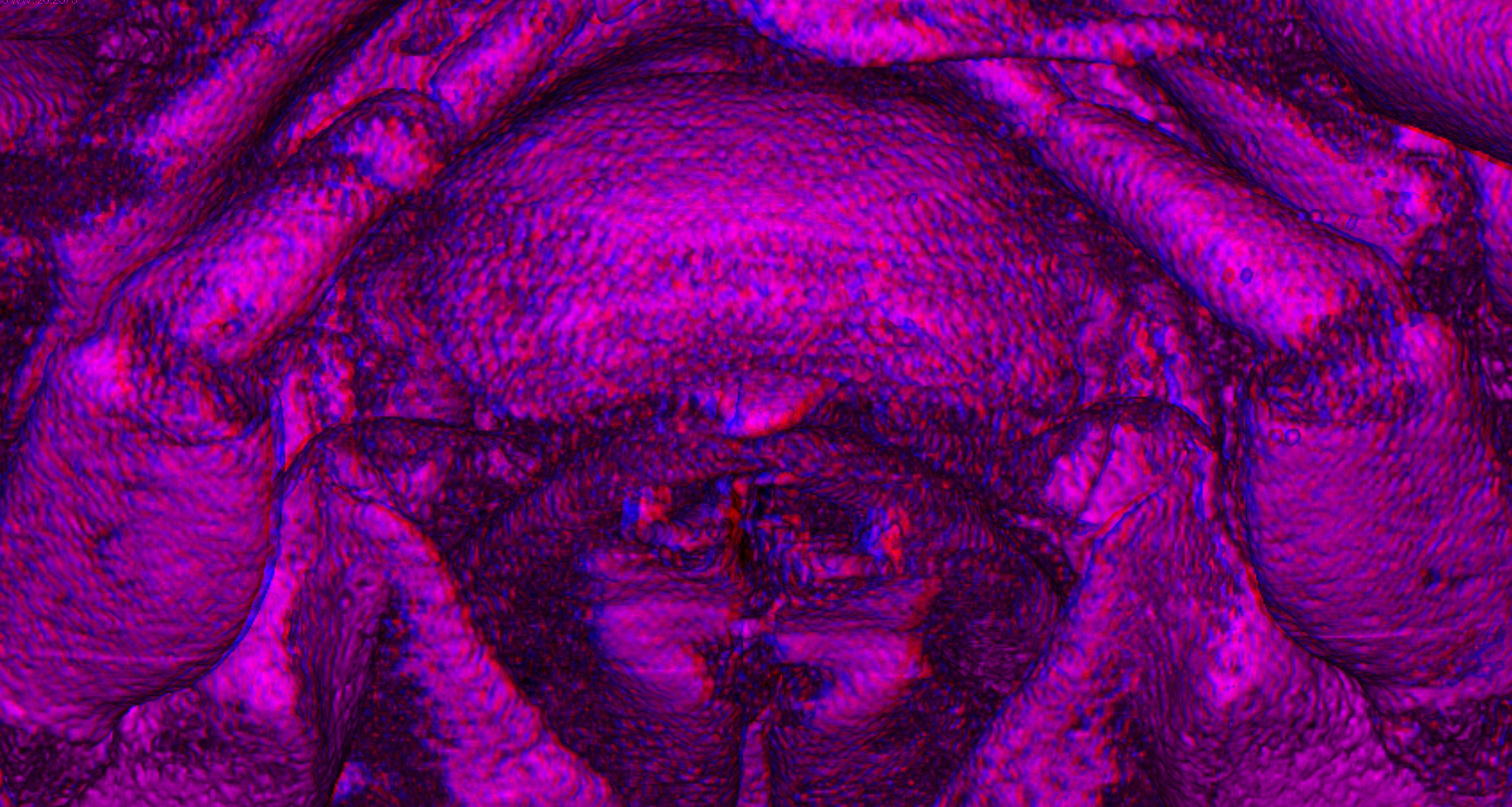

Supplement: Supplemental Information 4 [file peerj-04-2188-s004.png]

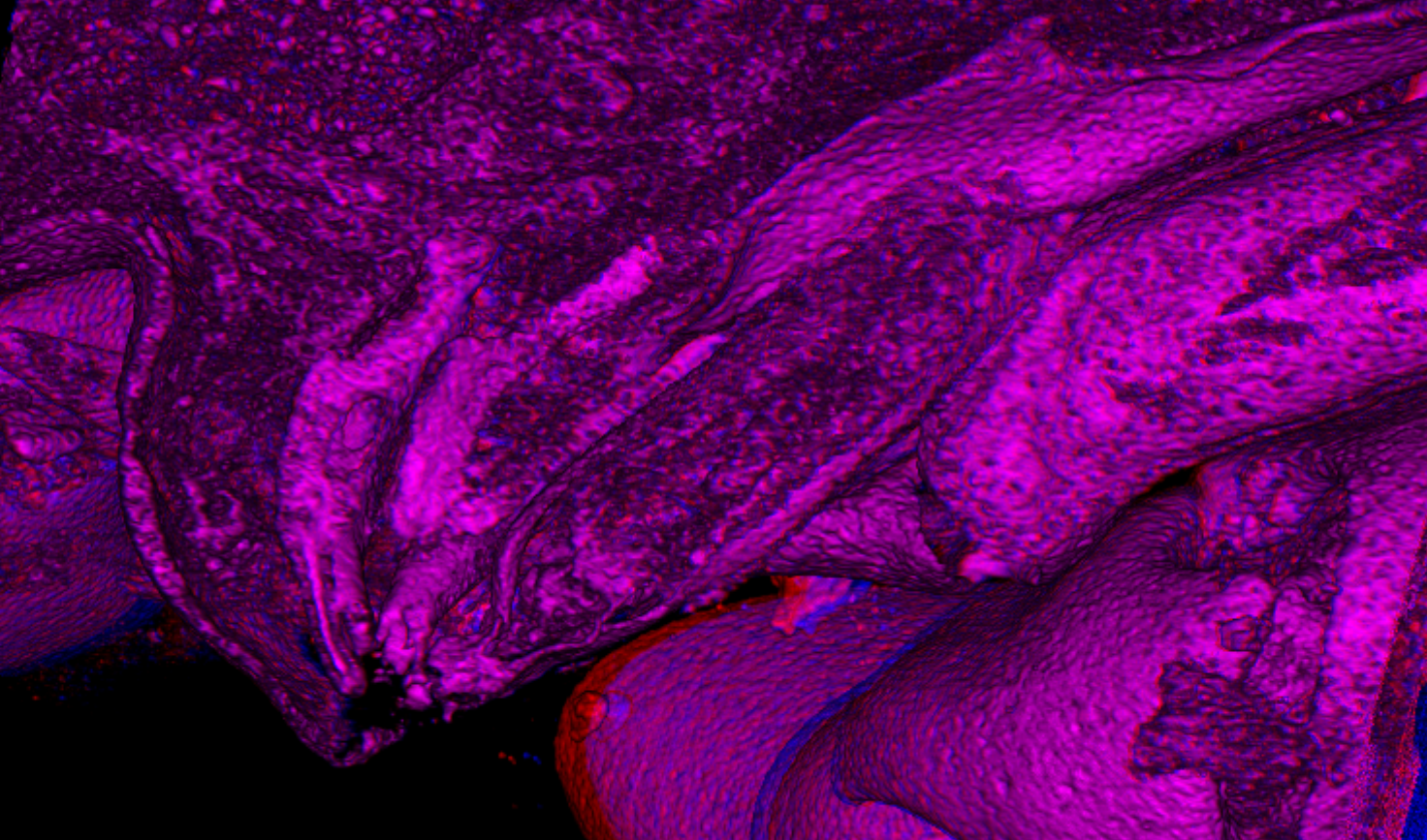

Supplement: Supplemental Information 5 [file peerj-04-2188-s005.png]

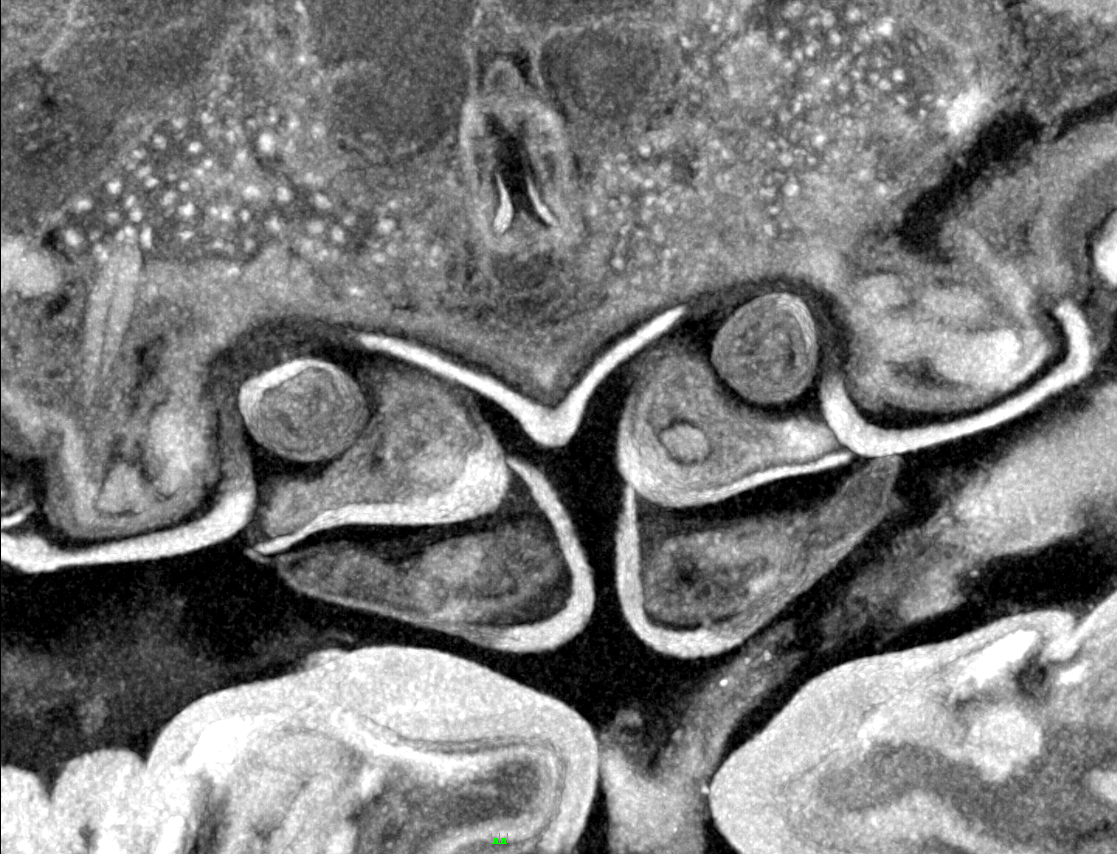

Supplement: Supplemental Information 6 [file peerj-04-2188-s006.png]

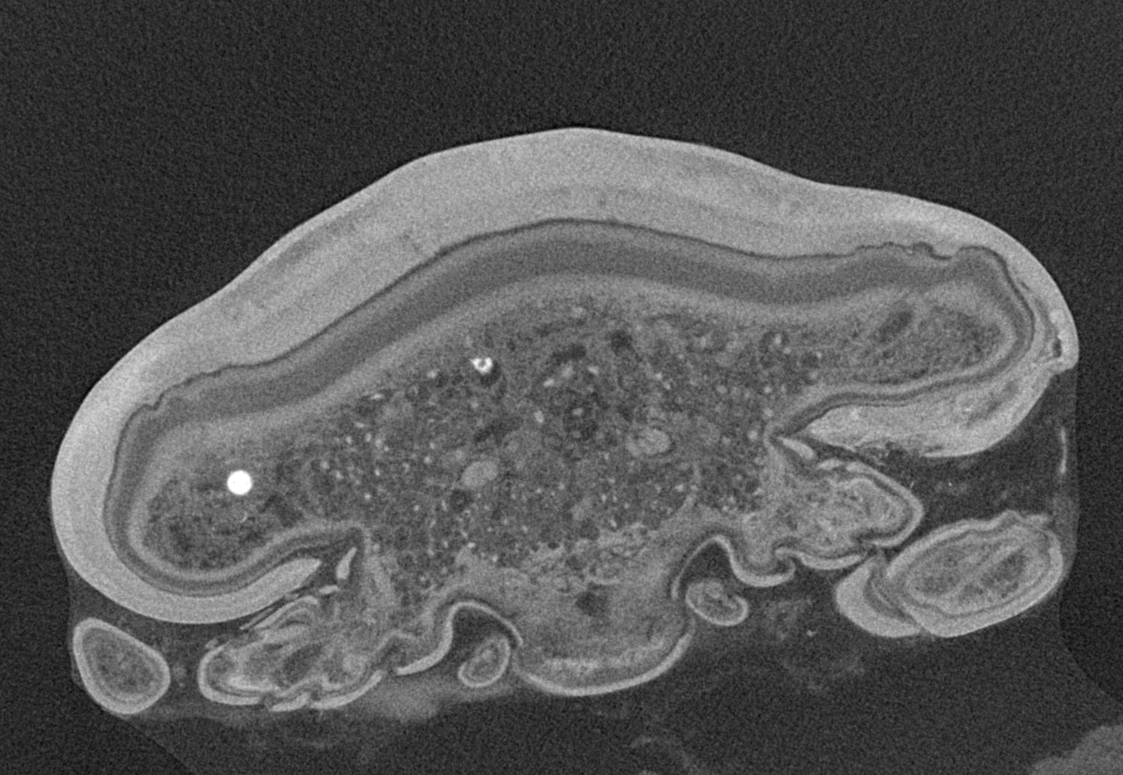

Supplement: Supplemental Information 7 [file peerj-04-2188-s007.png]

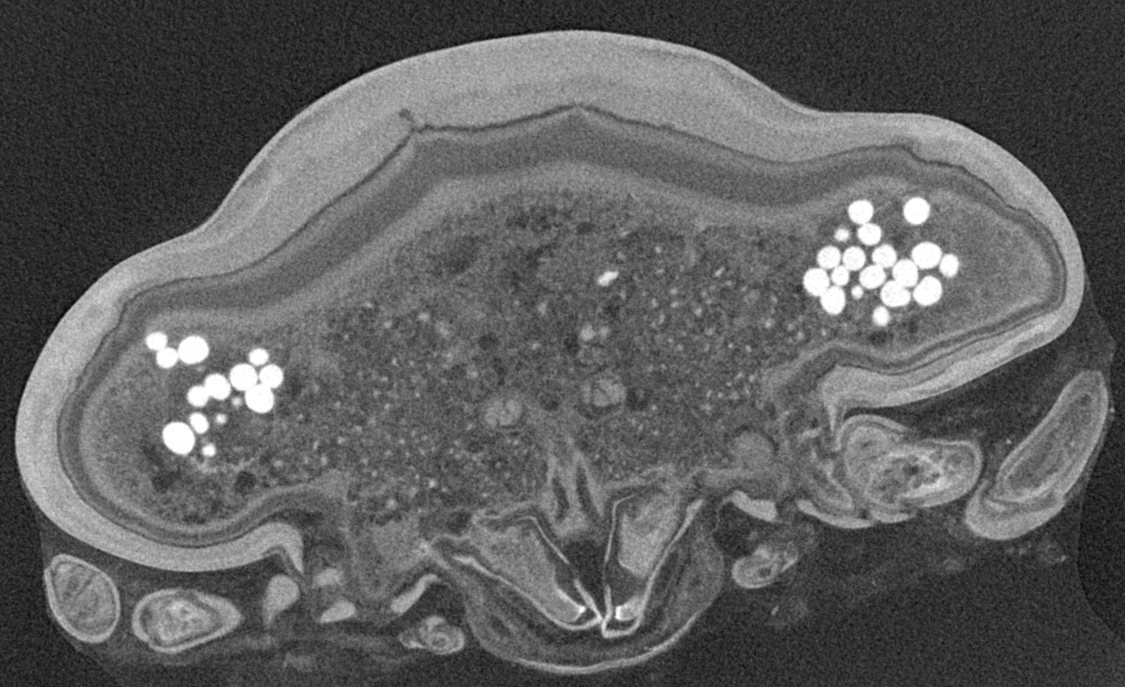

Supplement: Supplemental Information 8 [file peerj-04-2188-s008.png]

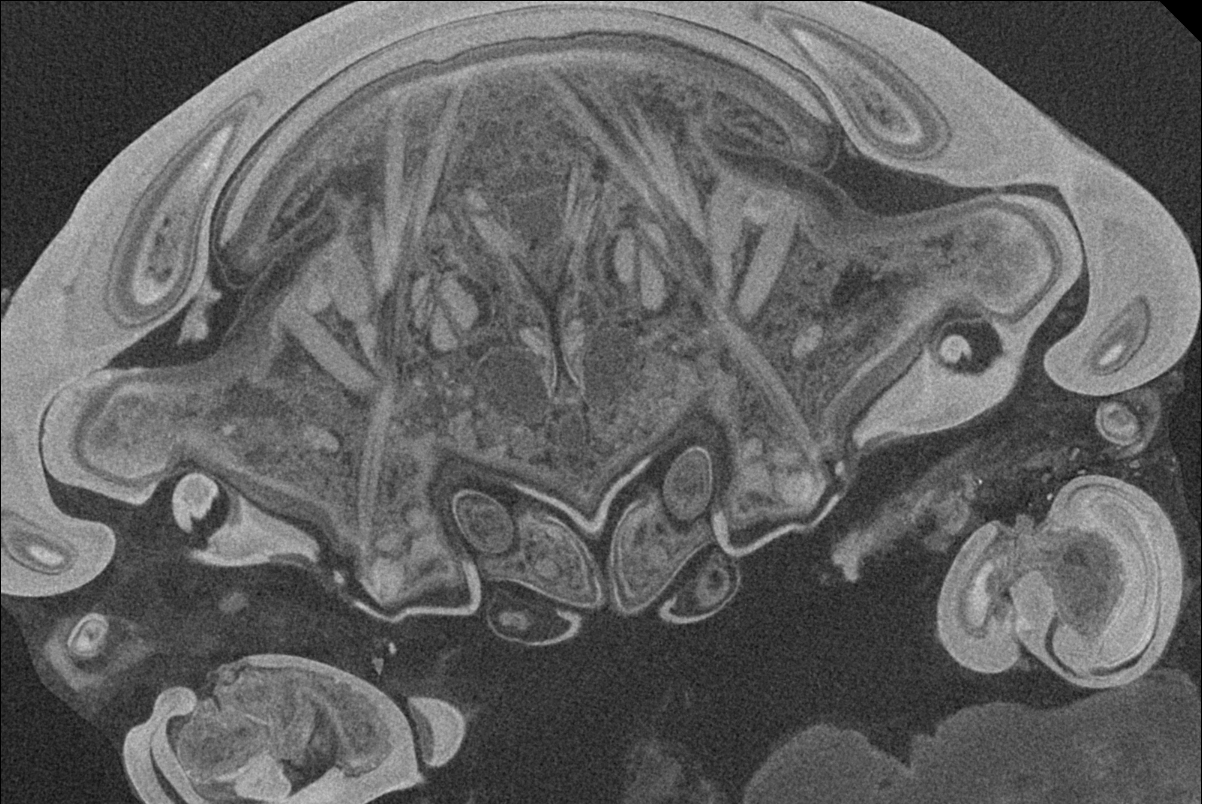

Supplement: Supplemental Information 9 [file peerj-04-2188-s009.png]

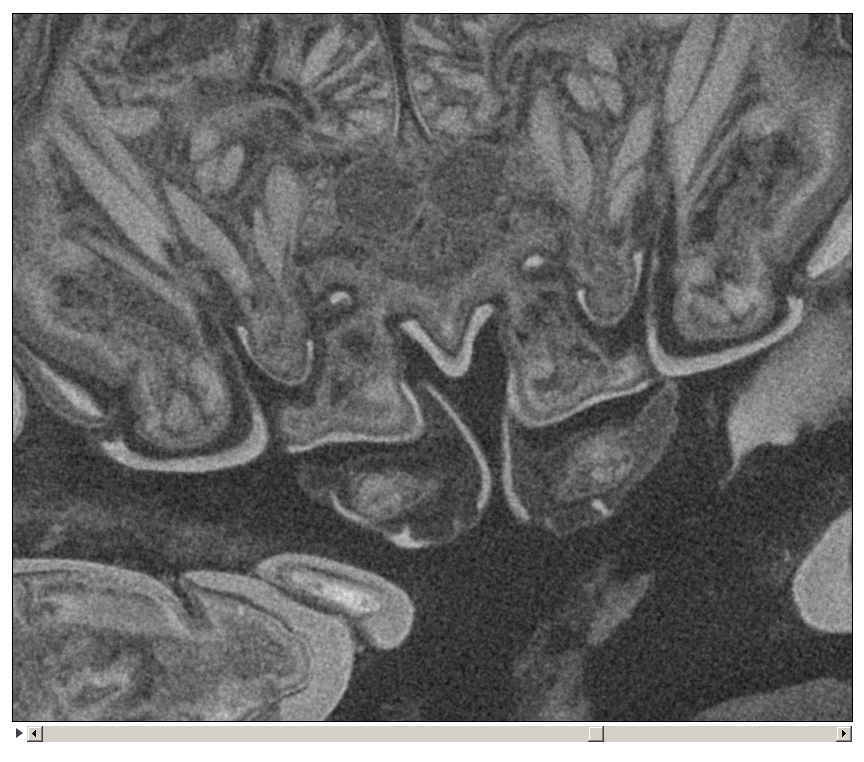

Supplement: Supplemental Information 10 [file peerj-04-2188-s010.png]

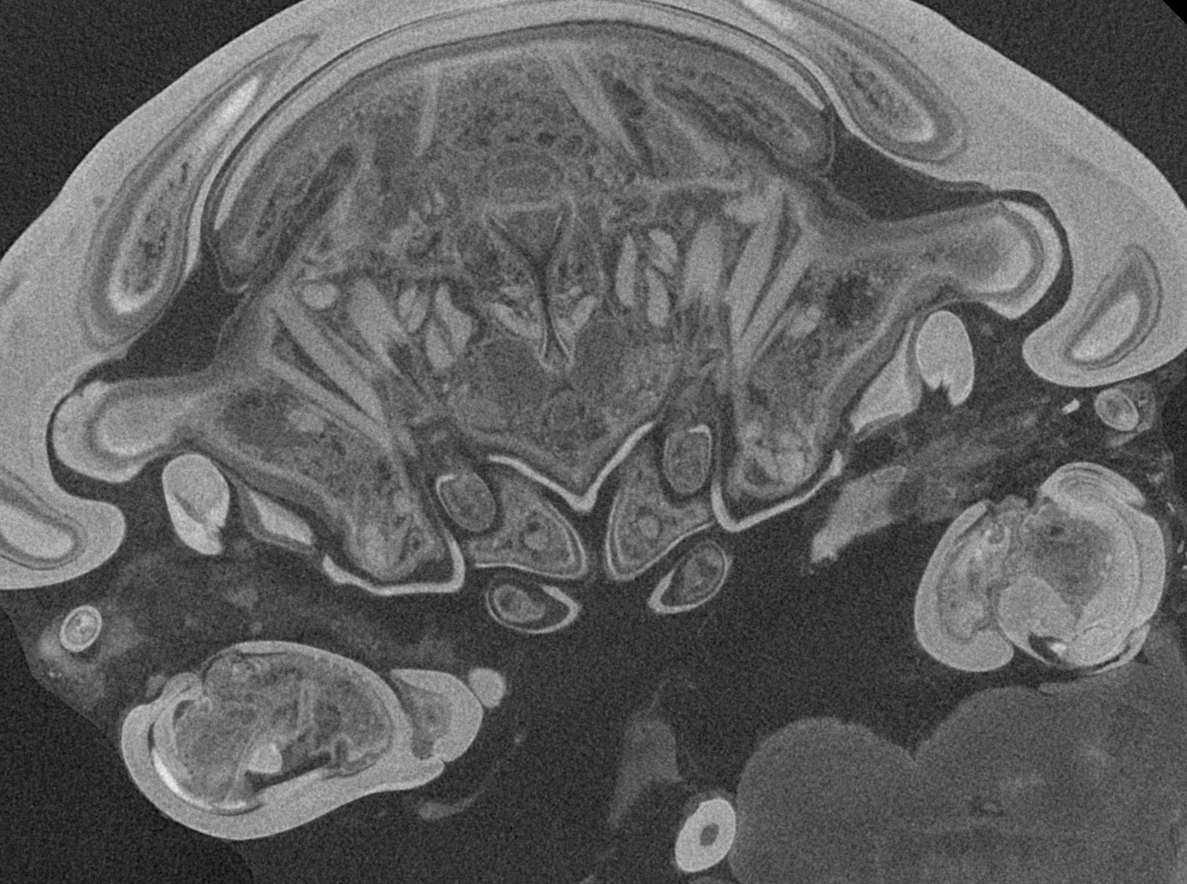

Supplement: Supplemental Information 11 [file peerj-04-2188-s011.png]

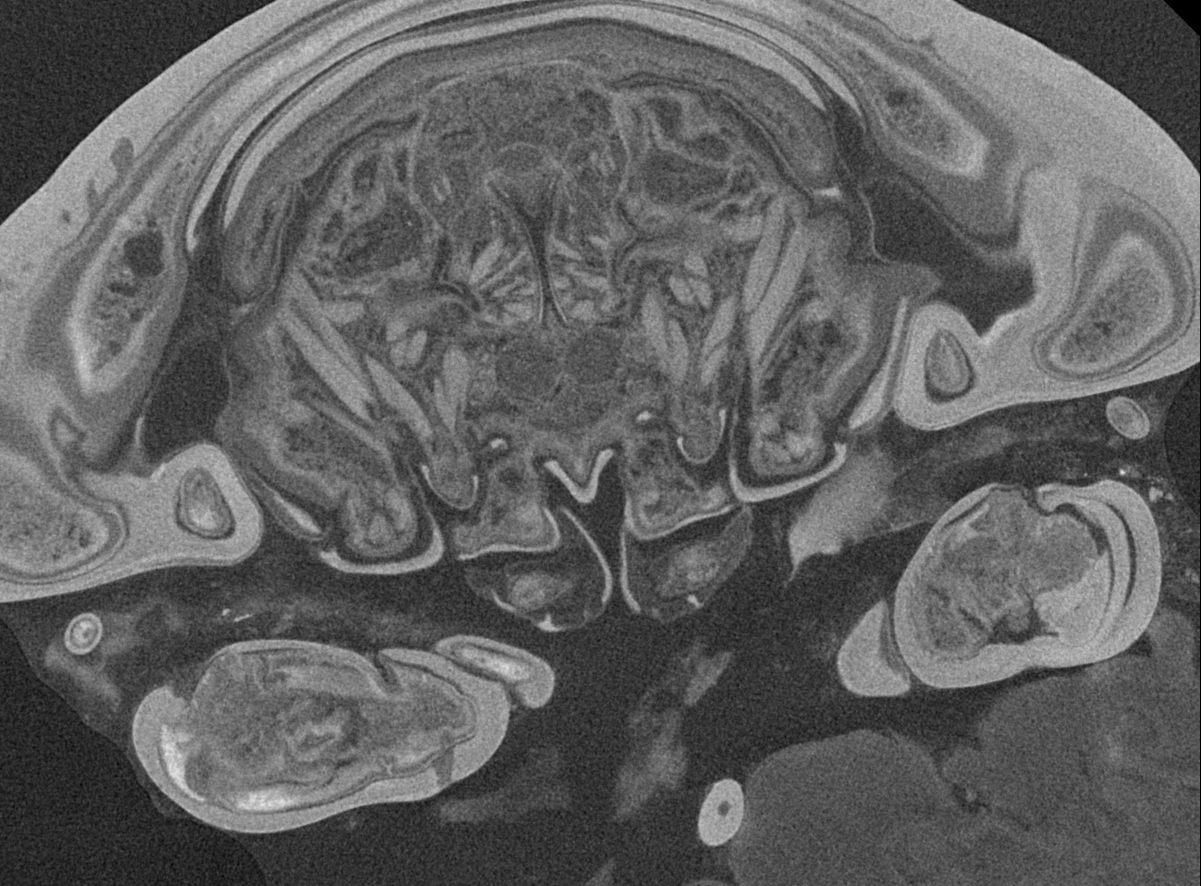

Supplement: Supplemental Information 12 [file peerj-04-2188-s012.png]

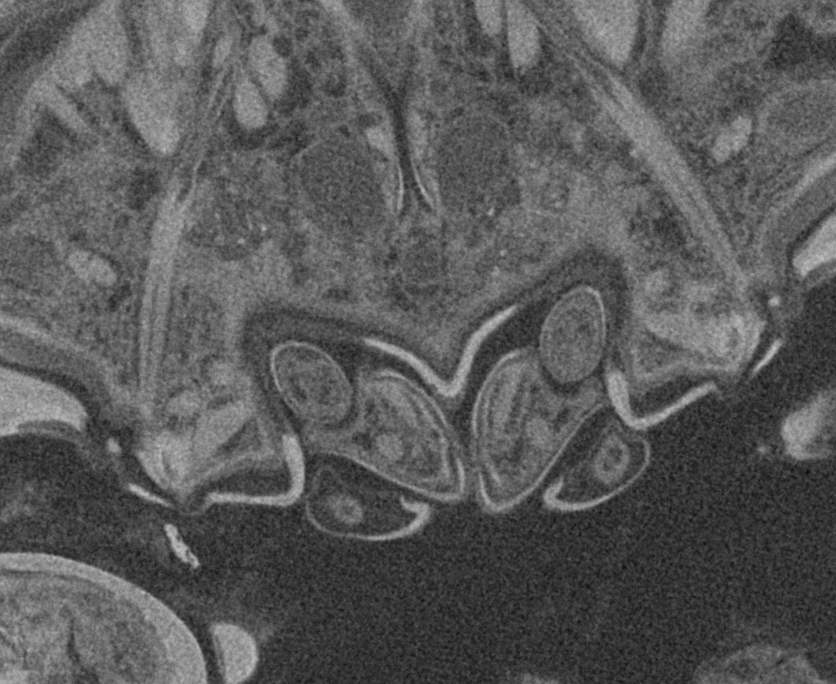

Supplement: Supplemental Information 13 [file peerj-04-2188-s013.png]

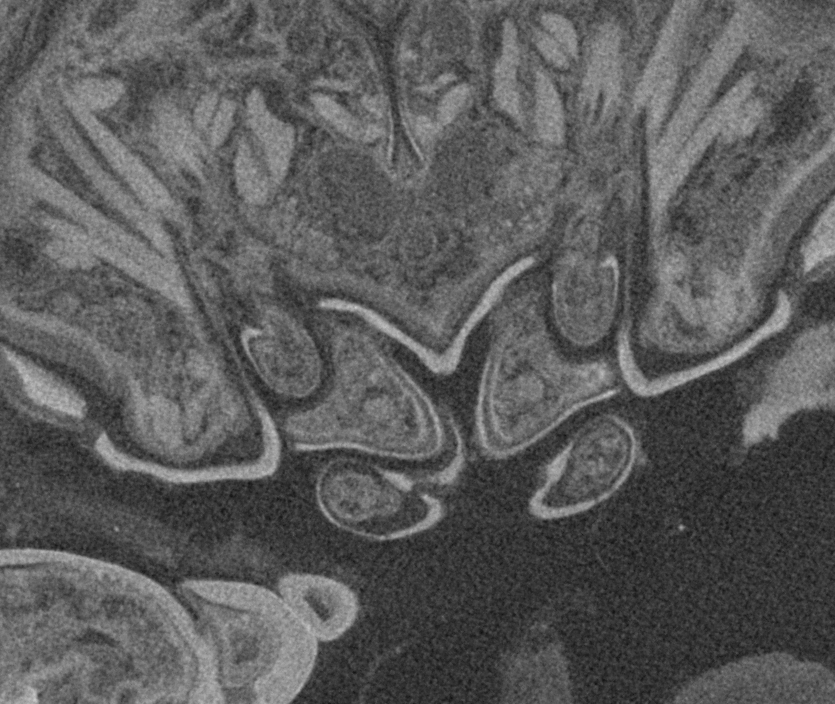

Supplement: Supplemental Information 14 [file peerj-04-2188-s014.png]

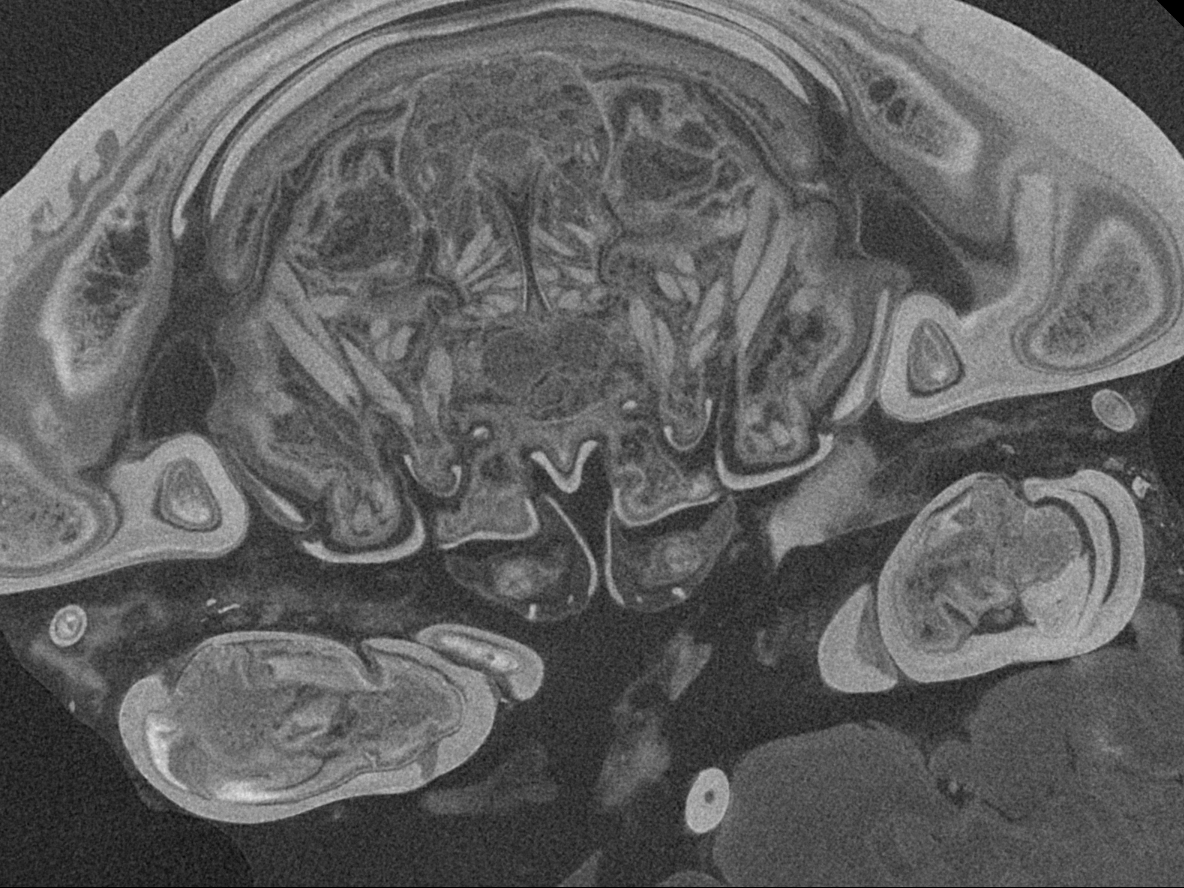

Supplement: Supplemental Information 15 [file peerj-04-2188-s015.png]
